# Pan-caner analysis identifies *PSMA7* as a targets for amplification at 20q13.33 in tumorigenesis

**DOI:** 10.1038/s41598-024-53585-0

**Published:** 2024-02-06

**Authors:** Guangying Sheng, Fuyu Li, Wen Jin, Kankan Wang

**Affiliations:** 1 State Key Laboratory of Medical Genomics, Ruijin Hospital Shanghai Institute of Hematology, National Research Center for Translational Medicine at Shanghai, Shanghai Jiao Tong University School of Medicine, 197 Ruijin Er Rd, Shanghai, 200025 China; 2 Ruijin Hospital, Sino-French Research Center for Life Sciences and Genomics, Shanghai Jiao Tong University School of Medicine, Shanghai, China; 3 School of Life Sciences and Biotechnology, Shanghai Jiao Tong University, Shanghai, China

**Keywords:** Cancer genomics, Gene expression, Genetic markers, Cancer, Biomarkers

## Abstract

The chromosome 20 long arm (20q) is one of the genomic hotspots where copy number alterations frequently occur in multiple types of tumors. However, it remains elusive which genes are implicated in 20q-related tumorigenesis. Here, by querying TCGA and GEO databases, we observed frequent copy number amplification at 20q and the chromosome subband 20q13.33 was amplificated in multiple cancers. Among those genes at 20q13.33, *PSMA7* was found with the strongest correlation with cancers. Further analysis revealed that *PSMA7* amplification was the most frequent genetic alteration event conferring adverse prognosis in various cancers. Consistent with the strong positive correlation between *PSMA7* amplification and gene expression, elevated *PSMA7* expression was observed in 20 of 33 types of cancers with a close link to adverse outcomes in certain tumors. In addition, *PSMA7* was essential for the growth of almost 1095 cancer lines. Mechanistically, aberrant *PSMA7* most probably influenced the proteasome and protease-related pathways to promote tumorigenesis and might be antagonized by several compounds, e.g., Docetaxel in relevant cancers. The current in-depth pan-cancer analysis refines our understanding of the crucial oncogenic role of copy number amplifications at *PSMA7* loci at the novel chromosome amplicon 20q13.33 across different tumors.

## Introduction

Copy number alterations (CNAs) are one of the types of genomic alterations that additionally consist of mutation (single nucleotide variation), chromosomal inversion, and translocation among others^1^. CNAs include amplifications (more copies, often focal), gains (a few additional copies, often broad), and deep and shallow deletions/losses (homozygous and heterozygous deletions)^2^. CNAs can impair genomic stability and dysregulate the involved genes to exert pro- or anti-tumor effects^2^. Previous scattered studies revealed the chromosome 20 long arm (20q) as one of the CNA hotspots harboring various cancer-related genes^3^. The 20q was frequently deleted or lost in hematological neoplasms^4^, while in ovarian cancer, upregulated genes and relevant aberrant DNA amplification were commonly concentrated at 20q^5^. Moreover, abnormal amplification of 20q was commonly reported in Barrett adenocarcinoma (BA+) tumor^6^ and colorectal^7^, breast^8^, prostate^9^, pancreatic^10^, and bladder^11^ cancers. Notably, Murty et al.^12^ found 20q11.2 and 20q13.13 as the novel chromosomal amplification loci carrying tumor-related genes in cervical cancer. Although these findings suggested critical roles of amplification at 20q in various tumors, it remained unclear which genes played a predominant role at 20q.

The proteasome subunit alpha-type 7 (*PSMA7*) encodes the alpha-type subunit of the 20S proteasome core complex from the 26S proteasome of the ubiquitin–proteasome system (UPS). By forming a regulatory structure “gateway” with other proteasome subunits, PSMA7 can modulate protein degradation^13^. Moreover, PSMA7 interacts with multiple important proteins including c-ABL^14^, hypoxia-inducible factor-1α (HIF-1α)^15^, hepatitis B virus (HBV) X (HBX) protein^16^, Rab7^17^, and endothelial monocyte-activating polypeptide 2 (EMAP2)^18^. Thanks to these interactions, PSMA7 is implicated in diverse cancer-related cellular activities, including transcription control, immunologic reaction, stress responses, cell cycle regulation, cellular differentiation, proliferation, and apoptosis. Additionally, c-ABL-mediated upregulation of PSMA7 led to the formation of alternative proteasome isoforms of PSMA7-PSMA7, eventually influencing cancer cell fitness^19,20^.

*PSMA7* overexpression has been observed in gastric^21,22^, cervical^12,23^, and testicular^24^ cancers and is shown to be closely linked to metastasis^25^, progression^26^, and reduced life span^27,28^ in certain cancers. Mechanistically, deficiency of *PSMA7* could inhibit cellular proliferation, migration, and invasion in colorectal^29^, gastric^22^, and breast^30^ cancers, and high-grade serous ovarian carcinoma (HGSOC)^31^. Besides, *PSMA7* depletion blunted the G_0_/G_1_ cell cycle and led to apoptosis of cervical cancer cells^23^. It was evidenced that the PSMA7-mediated proteasome degradation was essential to maintain cell survival of HGSOC^31^, cervical^23^, and castration-recurrent prostate cells^32^. Additionally, PSMA7 was found to contribute to tumorigenesis by activating the nuclear factor-κ-gene binding (NF-κB) pathway^33^ and the mitogen-activated protein kinase (MAPK) signaling pathway^22^, and interactions with miRNAs^30^. All these discoveries point to an oncogenic role of *PSMA7* in certain tumors. However, the precise roles and relevant mechanisms of *PSMA7* across human cancers remained less well understood.

Hereby, combining the cancer genome atlas (TCGA) and gene expression omnibus (GEO) data, we carried out a pan-cancer exploration of *PSMA7* at 20q concerning genetic alteration, survival state, gene expression, cellular pathway, molecular function, and potential drug screening, aiming to uncover the mechanisms of *PSMA7* in tumorigenesis and clinical outcomes across diverse human cancers.

## Results

### The amplification of chromosome 20q at the pan-cancer level

**Figure 1:**
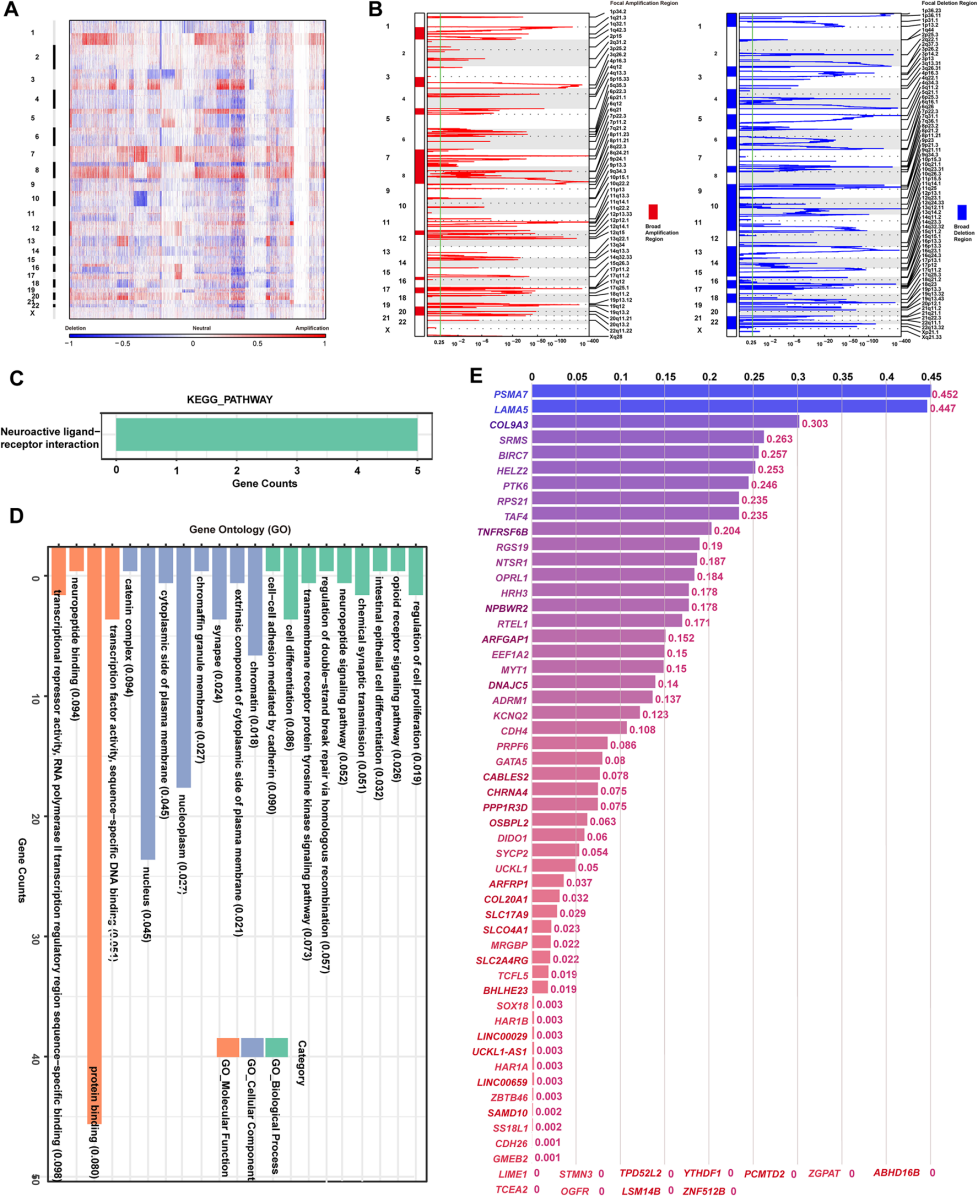
Copy number alterations (CNAs) of chromosome 20q across TCGA tumors. Using the revised Genomic Identification of Significant Targets in Cancer (GISTIC 2.0) pipeline, (**A**) the total segmented copy-number profile for each chromosome arm and (**B**) focal amplification and deletion events across the whole genome in 33 types of TCGA tumors were obtained and shown in a heatmap and detailed plots, respectively. Additionally, the broad CNA regions were marked as bars in the plots. Based on the gene set at 20q13.33, enrichment analysis of KEGG pathway (**C**) and GO (**D**) was conducted and shown as a bar chart with P values for each gene set attached in brackets. (**E**) The Phenolyzer was employed to evaluate the potential connection of “cancer” with each of the 85 genes at 20q13.33. A gene list ranked by scores was generated and visualized as bar plots.

To depict the genomic landscape of chr20 in human tumors, we employed the revised Genomic Identification of Significant Targets in Cancer (GISTIC2.0) pipeline^34–36^ to integrate the copy number raw data from 33 types of TCGA tumors (Fig. 1A, B, Supplementary Tabs. S1–S3). Overall, the statistical data for each chromosome arm revealed that the broad genomic amplification was more significant in the 20q than in the short arm of chr20 (20p) at the pan-cancer level (Fig. 1A, Supplementary Tab. S1). Consistently, this discovery was confirmed when the online SNP6 copy number analysis (GISTIC2) from Broad Firehose^35^ was run by each type of TCGA cancers in turn (Supplementary Fig. S1A). Besides, the GISTIC2.0-based pan-cancer integrated analysis detected only 3 focal events (amplification: 2; deletion: 1) located at chr20 (Fig. 1B, Supplementary Tabs. S2, S3), suggesting that amplification at chr20 was mostly in a broad level.

After downloading the data for CNA genes from the cBioPortal^37,38^, we queried the median frequency of amplification of mapped genes in tumor samples and further illustrated the amplification frequency in the corresponding chr20 subband (Table 1). Excluding chromosome subbands with less than 10 genes, the overall amplification frequency of 20q was 2.25%, which was significantly higher than that of 20p (0.8%). This finding highlighted the predominance of amplification in 20q (Fig. 1A, Supplementary Fig. S1A). Notably, 20q13.33, which involves 85 genes, displayed the highest alteration frequency of 2.7%, highlighting this region as a novel chromosome amplicon. Although amplification at this region was identified as a broad CNA event by pan-cancer integrated copy number analysis (Fig. 1B, Supplementary Tabs. S2, S3), the focal amplification of 20q13.33 was further found in 7 (of 30) tumor types of liver hepatocellular carcinoma (LIHC), lung adenocarcinoma (LUAD), ovarian serous cystadenocarcinoma (OV), rectum adenocarcinoma (READ), sarcoma (SARC), skin cutaneous melanoma (SKCM), and uterine corpus endometrial carcinoma (UCEC), according to detailed amplification plots generated by the online tool GISTIC2 from Broad Firehose (Supplementary Fig. S1B). Other relatively frequent chromosome subbands included 20q13.32, 20q13.2, and 20q13.31, with an amplification frequency of 2.6%, 2.6%, and 2.5%, respectively. 20q13.13 was another candidate amplicon with a relatively higher amplification frequency of 2.3% (Table 1), which had been identified in cervical cancer previously^12^. These findings revealed that amplification was a quite common genetic event for 20q, and 20q13.33 was likely to be an uncharted amplicon in human cancers.

**Table 1:** The median frequency of copy number amplification of each chr20 subband.

Cytband	Gene count	Median and range of frequency (%)	P value
20q	415^a^	2.25 (1.4–3.4)^b^	< 0.0001^c^
20q11	1	3.1	NA
20q13.33	85	2.7 (2.0–2.9)	Reference
20q13.32	19	2.6 (2.3–2.7)	0.0063
20q13.2	19	2.6 (2.4–3.2)	0.8792
20q13.31	16	2.5 (2.2–2.7)	< 0.0001
20q13.2-q13.31	1	2.5	NA
20q13.32-q13.33	1	2.4	NA
20q11.22-q11.23	1	2.4	NA
20q13.13	38	2.3 (1.8–2.6)	< 0.0001
20q11.21-q11.22	1	2.3	NA
20q11.22	43	2.2 (1.9–2.6)	< 0.0001
20q13.12	87	2.1 (1.9–2.5)	< 0.0001
20q11.21	49	2.0 (2.1–3.4)	0.0220
20q12-q13.11	1	2	NA
20q13.11	2	2	NA
20q11.23	49	1.7 (1.5–2.1)	< 0.0001
20q11.23-q12	1	1.5	NA
20q12	10	1.45 (1.4–1.5)	< 0.0001
20q11.1	1	1.15 (0.7–2.6)	NA
20p	216^a^	0.8 (0.6–1.5)^b^	< 0.0001^c^
20p12.3-p12.2	1	1	NA
20p13	94	0.9 (0.8–1.1)	< 0.0001
20p12.3	23	0.9 (0.8–1.1)	< 0.0001
20p11.21	36	0.8 (0.7–1.0)	< 0.0001
20p12.1	21	0.8 (0.6–1.5)	< 0.0001
20p12.2	13	0.8 (0.8–0.9)	< 0.0001
20p11.1	5	0.8 (0.8–1.1)	NA
20p13-p12.3	1	0.8	NA
20p11.23	29	0.7 (0.6–0.8)	< 0.0001
20p11.22	8	0.7 (0.7–0.8)	NA

(a) Total count of the mapped genes; (b) The median and range of amplification frequency of the mapped genes represented the amplification frequency of the corresponding subband at the long (q) or short arm (p) of chromosome 20. The chromosome subbands with a gene count below 10 were excluded. (c) chr20p vs. chr20q.

NA, not available.

*P* value less than 0.05 is statistically significant.

To investigate which pathways were mainly involved in the 20q13.33-amplified cancers, we conducted an enrichment analysis of the 85 genes at 20q13.33 (Fig. 1C, D). The Kyoto Encyclopedia of Genes and Genomes (KEGG) and gene ontology (GO) data manifested that most genes were significantly enriched in multiple cancer-related cellular and molecular functions, especially of translocations between the nucleus, nucleoplasm, and chromatin, cell proliferation, and differentiation (Fig. 1D). However, it remained undetermined which gene primarily mediated the effects of amplified 20q13.33 on cancer initiation and development. Thus, we used the Phenolyzer^39^ tool to evaluate the potential association of the 85 genes at 20q13.33 with cancer development. Notably, *PSMA7*, one of the proteasome family members, achieved the highest correlation score of 0.452 with cancer (Fig. 1E), suggesting the crucial oncogenic role of *PSMA7* in 20q13.33. We thus focused on *PSMA7* for further study.

### *PSMA7* gene amplification occurs in multiple types of tumors.

**Figure 2:**
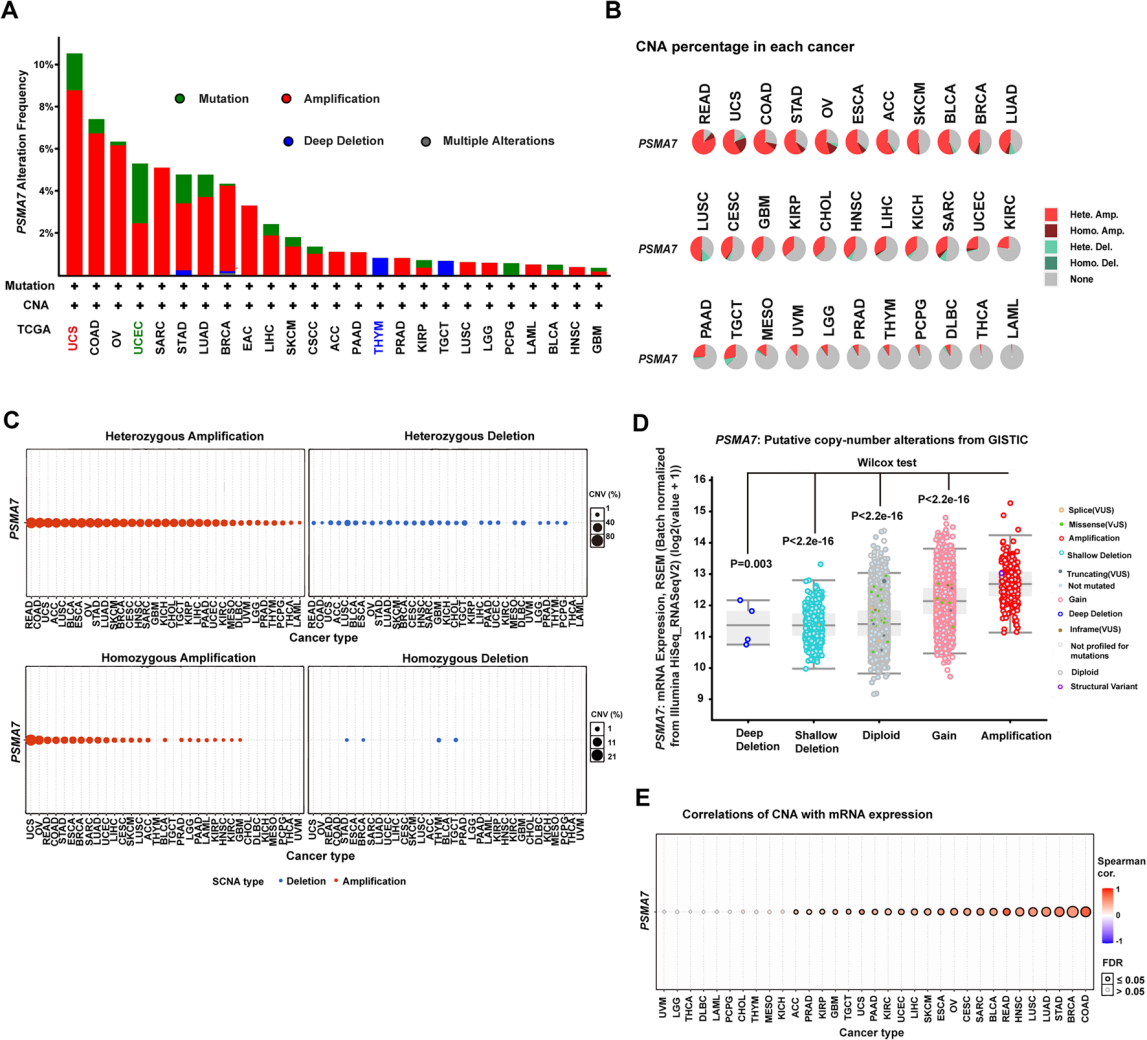
Copy number alteration of *PSMA7* across diverse TCGA cancers. (**A**) Via the cBioPortal web, the genetic variation incidence and mutation type of *PSMA7* were investigated across different tumors of TCGA. The percentage (**B**) and genotype status (**C**) of *PSMA7* CNA were also explored by the TCGA-based GSCA approach. (**D**) Based on the copy number data of GISTIC databases, the cBioPortal web was employed to evaluate the *PSMA7* expression level represented by different types of putative CNA. (**E**) Through the GSCA tool, the potential link of *PSMA7* CNA to gene expression was measured across TCGA tumors.

To comprehensively explore the genetic alterations of the *PSMA7* gene locus rather than CNA^1,2^, we conducted a genetic alteration survey of *PSMA7* across 32 types of TCGA cancers via the cBioPortal approach. The genetic alterations of *PSMA7* were found in a total of 25 types of tumors, and amplification was the most frequent molecular alteration observed in *PSMA7* (Fig. 2A). The uterine carcinosarcoma (UCS) cases carried the utmost amplification incidence of *PSMA7* (~ 8.8%), followed by colon adenocarcinoma (COAD) (~ 6.7%) and OV (~ 6.2%). Finally, we discovered that cohorts with thymoma (THYM) harbored deep deletion of *PSMA7* with the highest frequency (0.81%) but represented a minority of cancers.

Consistent with the above results, according to** t**he Gene Set Cancer Analysis (GSCA) (a TCGA-based tool)^40^, the CNA of *PSMA7* was the major genetic alteration event observed in 33 types of TCGA tumors (Fig. 2B). The highest CNA frequency appeared in nearly 95% of patients with READ. In addition, the amplified CNA of *PSMA7* predominated in each type of tumor with a frequency of up to 80%, while the copy number deletion was most frequently observed in less than 15% of patients with lung squamous cell carcinoma (LUSC). Furthermore, both amplification and deletion of CNA occurred in heterozygous status, with the highest frequency of 80% in cases with READ (Fig. 2C). In contrast, the homozygous CNA was most commonly observed in only 21% of patients with UCS (Fig. 2C). These data indicated that heterozygous copy number amplification of *PSMA7* represented the major genetic variation event across the TCGA tumors.

### *PSMA7* amplification exhibits a positive link to gene expression

Emerging evidence supported that genomic alterations could alter the transcript levels of mapped genes to promote cancer development and progression^2^. Thus, we asked whether copy number amplification of *PSMA7* affected its gene expression levels. Based on the GISTIC database, the *PSMA7* expression level in the amplification of putative CNA was significantly higher than that in other types of CNA (Fig. 2D). Besides, spearman correlation analysis via the GSCA web also documented that the *PSMA7* CNA exhibited a positive link to gene expression in 23 of 33 types of TCGA tumors with false discovery rate (FDR) ≤ 0.05 (Fig. 2E). The correlations in detailed cancers were displayed inSupplementary Fig. S2, especially 0.8 in READ and 0.78 in COAD. Collectively, these data suggested that copy number amplification of *PSMA7* might lead to gene overexpression in human cancers.

### Differentially expressed *PSMA7* between cancer and normal tissues

**Figure 3:**
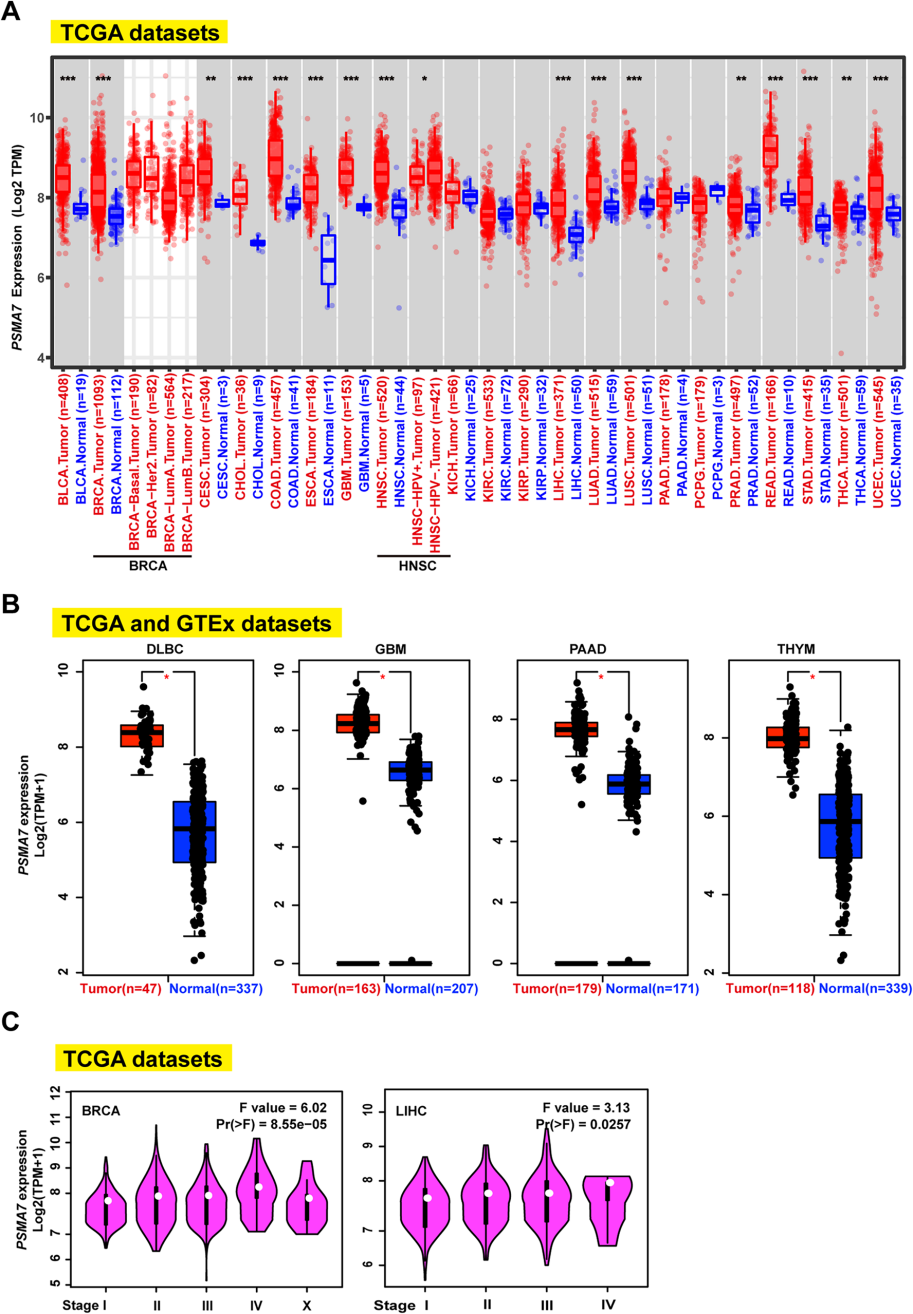
Expression level of *PSMA7* in various cancers. (**A**) Via the TIMER2 web, the *PSMA7* expression level was evaluated across diverse tumors or certain subtypes with matching normal samples from the TCGA dataset. * P ≤ 0.05; ** P ≤ 0.01; *** P ≤ 0.001. (**B**) The GEPIA2 was employed to generate the box plots data for the expression level of *PSMA7* across DLBC, GBM, PAAD, and THYM, with the matched healthy samples from the GTEx dataset being added into the control cohorts. * P ≤ 0.01. (**C**) Based on the logarithmic scale of Log2 (TPM + 1), *PSMA7* expression in each pathological stage of BRCA and LIHC was evaluated by GEPIA2.

Having confirmed a close positive association of copy number amplification of *PSMA7* with gene expression (Fig. 2D, E, Supplementary Fig. S2), we further verified whether *PSMA7* was overexpressed in diverse TCGA cancers, contrasting with healthy volunteer samples via the tumor immune estimation resource 2 (TIMER2)^41^. In doing so, elevated *PSMA7* expression was confirmed in the cancers of bladder urothelial carcinoma (BLCA), breast invasive carcinoma (BRCA), cholangiocarcinoma (CHOL), COAD, esophageal carcinoma (ESCA), glioblastoma multiforme (GBM), head and neck squamous cell carcinoma (HNSC), LIHC, LUAD, LUSC, READ, stomach adenocarcinoma (STAD), UCEC, and cervical squamous cell carcinoma and endocervical adenocarcinoma (CESC), prostate adenocarcinoma (PRAD), thyroid carcinoma (THCA), as compared with the matched control tissues (Fig. 3A).

Furthermore, adding the healthy samples of the genotype-tissue expression (GTEx) dataset to the current control group, we observed significantly overexpressed *PSMA7* in tumor samples of diffuse large B-cell lymphoma (DLBC), GBM, pancreatic adenocarcinoma (PAAD), and THYM (Fig. 3B), but not in other tumors, including adrenocortical carcinoma (ACC), CESC, brain lower-grade glioma (LGG), OV, SKCM, testicular germ cell tumors (TGCT), or UCS (Supplementary Fig. S3A) by the gene expression profiling interactive analysis 2 (GEPIA2)^42^. Moreover, *PSMA7* expression showed significant differences across the pathological stages of BRCA and LIHC while not including other tumors (Fig. 3C, Supplementary Fig. S3B). Taken together, these data showed that *PSMA7* overexpression frequently occurred in most human cancers.

### PSMA7 amplification is an adverse prognostic factor in most tumors.

**Figure 4:**
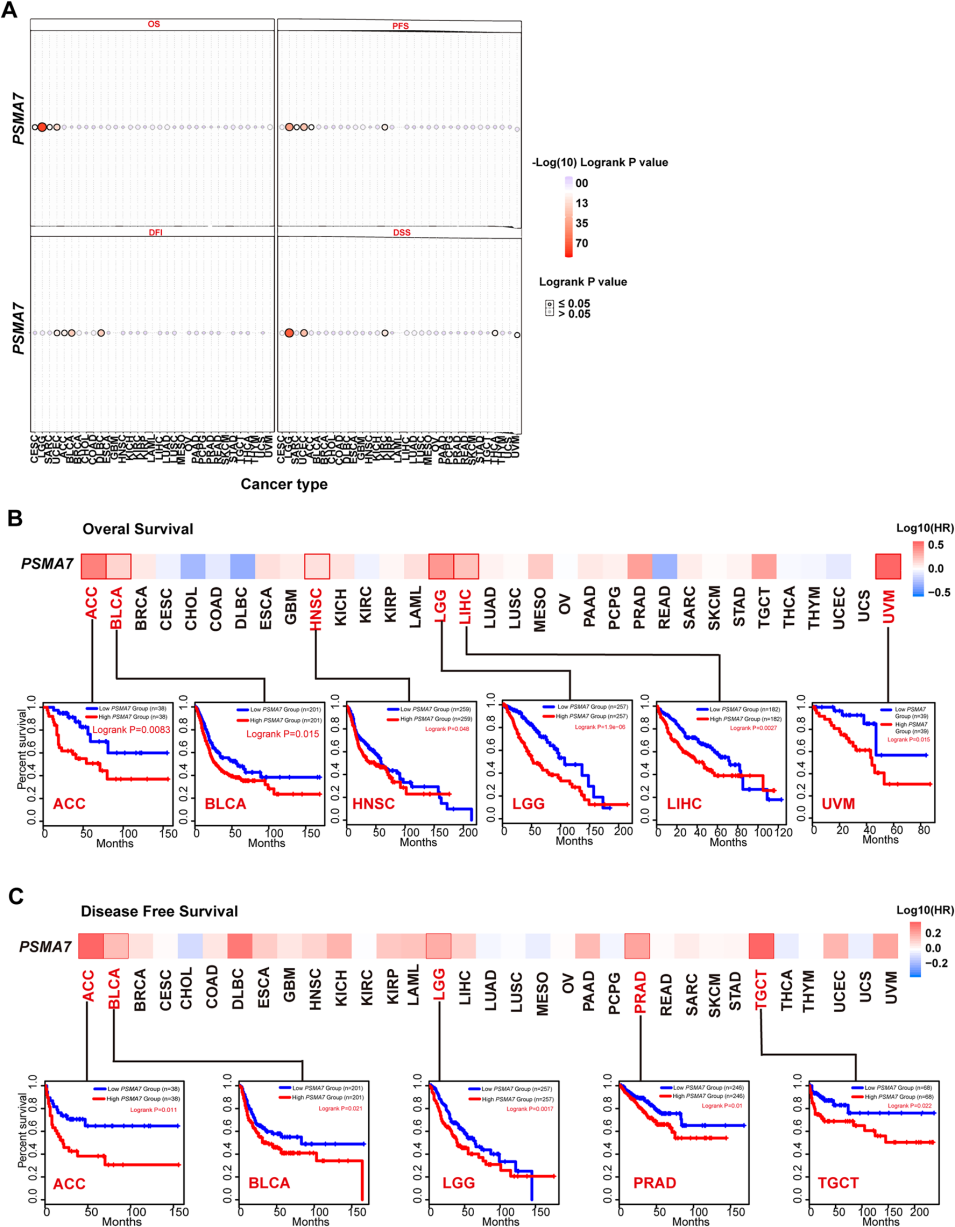
The prognostic significance of *PSMA7* CNA and expression on diverse TCGA tumors. (**A**) By the log-rank test, the prognostic significance of *PSMA7* CNA on TCGA tumors was estimated for OS, PFS, DSS, and DFI. (**B**, **C**) The potential link of *PSMA7* expression to OS and DFS prognosis for numerous TCGA cancers was investigated through the approach GEPIA2. The positive results were displayed as survival maps and K-M plots.

The prognostic significance of *PSMA7* CNAs (amplification, deletion, and wild type) was evaluated by log-rank survival analysis using the TCGA dataset (Fig. 4A, Supplementary Fig. S4). The kidney renal clear cell carcinoma (KIRC) patients harboring CNA amplification achieved shorter progression-free survival (PFS) and disease-specific survival (DSS) than those without CNA. Similarly, a reduced median time of DSS was observed in patients with THCA and uveal melanoma (UVM) with amplification. Nevertheless, the highly amplified CNA of *PSMA7* was linked to better PFS and disease-free interval (DFI) for ACC. In contrast, the amplified CNA of *PSMA7* was associated with worse PFS, DSS, DFI, and overall survival (OS) prognosis for UCEC. Patients with LGG with amplification had a shorter median time of OS, PFS, and DSS, and those with SARC with amplified CNA suffered much shorter OS and PFS times when compared with cases without variations. The amplified CNA of *PSMA7* was related to worse DFI for BLCA and lymphoid neoplasm DLBC, but similar OS for CESC. The above results showed that copy number amplification of *PSMA7* was a predominant genetic alteration event with adverse outcome effects on most TCGA tumors.

To evaluate whether the amplification of *PSMA7* represented an independent prognostic factor in the above tumors, we introduced a multivariate Cox regression model to adjust for established clinical variables such as patient age, tumor stage, and histological grade (Supplementary Tabs. S4-S8). Using survival data from Genomic Data Commons^43^, we found that amplification of *PSMA7* achieved a relatively higher hazard ratio (HR) with all P values below 0.01 in OS, progression-free interval (PFI), and DSS of LGG, respectively, compared to diploid. However, this statistical trend remained significant merely for OS of LGG after isocitrate dehydrogenase (*IDH*) mutation and 1p/19q co-deletion were enrolled. Similarly, the amplified *PSMA7* exhibited significant HRs exceeding 1 for OS and DSS in patients with SARC, contrasting to those with normal diploids. Notably, the OS of THCA showed the highest HR of 44.04 for amplified *PSMA7* with statistical significance when patient age was a continuous variable. In addition, we also observed an HR below 1 with a P value less than 0.05 for amplification of *PSMA7* in PFI of KIRC, but no positive data for common survival indicators of UCEC, BLCA, and ACC (data not shown). These findings further suggested that amplification of *PSMA7* might be an independent adverse biomarker for survival in certain types of tumors.

### The overexpressed *PSMA7* is linked to adverse clinical outcomes in tumor patients

Next, we employed GEPIA2 to investigate the prognostic significance of *PSMA7* overexpression across numerous cancers according to the TCGA and GEO projects. The overexpressed *PSMA7* displayed a considerable link to the bad OS of ACC, BLCA, HNSC, LGG, LIHC, and UVM in the TCGA cohorts (Fig. 4B). Additionally, elevated *PSMA7* expression showed an association with reduced disease-free survival (DFS) in the TCGA samples of ACC, BLCA, LGG, PRAD, and TGCT (Fig. 4C).

Furthermore, the Kaplan–Meier (K-M)^44^ plotter-based survival analysis was carried out to reveal the influence of high *PSMA7* expression on the clinical prognosis of breast, ovarian, lung, gastric, and liver cancers (Supplementary Fig. S5A–E). As expected, breast cancer patients with elevated *PSMA7* expression exhibited poor OS, relapse-free survival (RFS), and DFS. Similarly, overexpressed *PSMA7* showed a connection with adverse OS but not PFS or post-progression survival (PPS) for ovarian cancer, whereas poor OS and PPS but good first progression (FP) for lung carcinoma. In gastric cancer, elevated *PSMA7* expression merely exhibited a significant link with inferior FP. Moreover, the upregulated transcript level of *PSMA7* had a close link to poor OS and RFS while not PFS or DFS for liver cancer. The meta-analysis of these survival data further confirmed that *PSMA7* overexpression was an adverse factor for breast, gastric, and liver cancers but not ovarian or lung cancers (Supplementary Fig. S6). Additionally, consistent results were shown in survival analyses by subgroup when detailed clinical factors were considered (Supplementary Tabs. S9–S13). These aforementioned discoveries suggested that highly expressed *PSMA7* represented a poor prognostic biomarker for most human tumors.

### Abnormal *PSMA7* adversely influences genomic DNA stability

As an initiating step of tumorigenesis, genomic instability (GIN) can induce various cancer-related pathological processes such as DNA damage reaction defects, cell cycle checkpoint dysfunction, and oncogene activation^45^. The GIN can be measured by two biological indicators, microsatellite instability (MSI)^46^ and tumor mutational burden (TMB)^47^. In particular, the TMB has become a reliable biomarker to predict the response to programmed death 1 and ligand 1 (*PD-1/PD-L1*) therapy for cancers^47^. In this regard, we attempted to explore the possible link of *PSMA7* expression to TMB and MSI across TCGA tumors. Consistent with the expected result, *PSMA7* expression exhibited a positive relationship with MSI of KIRC, DLBC, TGCT, kidney renal papillary cell carcinoma (KIRP), LIHC, UCEC, and BLCA (Supplementary Fig. S7A), pointing to compromised DNA mismatch repair function in these tumors. Besides, *PSMA7* expression also harbored a positive association with TMB in most types of tumors, including SARC, PAAD, BRCA, LGG, LUAD, STAD, SKCM, ACC, OV, LIHC, LUSC, PRAD, and BLCA (Supplementary Fig. S7B). Collectively, we postulated that aberrant high expression of *PSMA7* might result in decreased genomic stability in a general way and serve as a predictive immunotherapy biomarker.

Next, we wondered why *PSMA7* alterations imposed such a great negative influence on genomic stability. Since amplification of *PSMA7* accounted for predomination in genetic alteration types, we compared the alteration frequency of genes in 276 cases with CNA-amplified *PSMA7* and that in 10,684 cases without the corresponding *PSMA7* abnormality in TCGA tumors (Supplementary Fig. S8A). Interestingly, among 373 differential genes with an alteration frequency over a 5.5 log ratio, 122 (32.71%) genes preferentially were centralized at chromosome 20 (Supplementary Fig. S8B). Further analysis revealed that 84 of 122 (68.25%) genes were mostly enriched in the region of chromosome 20q13.33 where *PSMA7* was exactly located (Supplementary Fig. S8C). The top 30 genes ranked by the frequency at 20q13.33 were visually displayed in Supplementary Fig. S8D. In contrast to genes in samples without amplified *PSMA7*, most of those in cases with *PSMA7* amplification experienced high alteration burdens, with frequencies of nearly 80%. These discoveries suggested again that 20q13.33 harboring *PSMA7* among others was plausibly a novel amplicon that frequently existed in human cancers. In addition to 20q13.33, we also found other chromosomal amplification sites, such as 20q13.32, 20q13.31, and 20q13.2 (Supplementary Fig. S8B), corresponding to the potential chr20 amplicons ranked by amplification frequency in Table 1. The results manifested a co-occurrence amplification pattern of *PSMA7* and other 20q-related genes that together contributed to GIN.

### The underlying molecular mechanism of aberrant *PSMA7* in tumorigenesis

**Figure 5:**
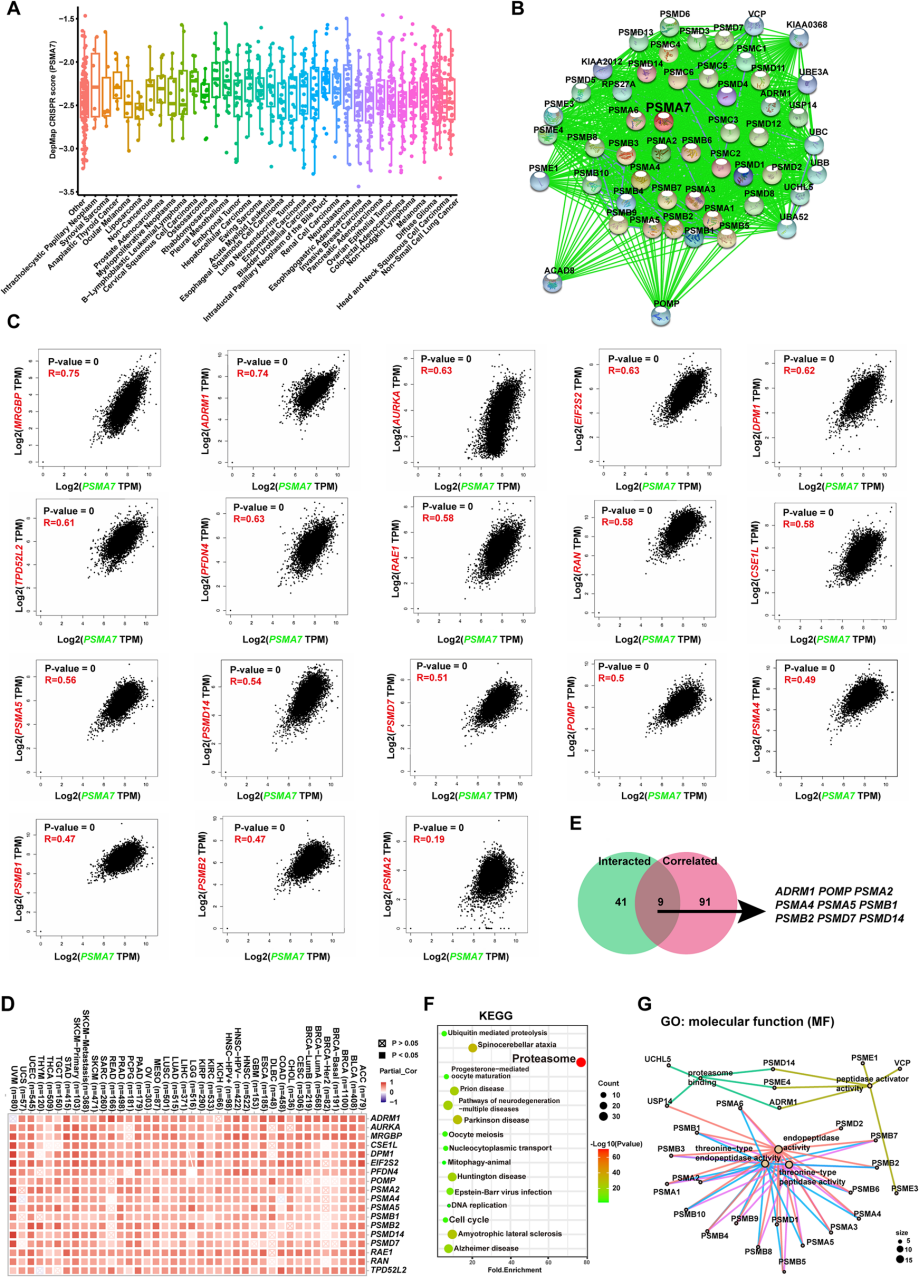
The underlying molecular mechanism behind the aberrant *PSMA7* in tumorigenesis. (**A**) The dependency of tumor cell viability on *PSMA7* was evaluated by the whole-wide CRISPR knockout screens in the 1095 cancer cell lines from the Depmap database. (**B**) Via STRING, we acquired proteins with experimental validation to interact with PSMA7. (**C**) According to the TCGA tumor dataset, we found the first 100 genes linked to *PSMA7* expression through the GEPIA2 approach. Further, the scatter plot displayed the expression correlations of *PSMA7* with 18 selected genes of *MRGBP*, *ADRM1*, *AURKA*, *EIF2S2*, *DPM1*, *TPD52L2*, *PFDN4*, *RAE1*, *RAN*, *CSE1L*, *PSMA5*, *PSMD14*, *PSMD7*, *POMP*, *PSMA4*, *PSMB1*, *PSMB2*, and *PSMA2*. (**D**) The corresponding validation data across diverse TCGA cancers were generated into a heatmap by TIMER2.0. (**E**) The shared proteins between the PSMA7-interacting and expression-related genes were discovered by the interactive Venn diagram viewer Jvenn. (**F-G**) The KEGG and GO analysis of the aforementioned two gene sets (**B** and **C**) was conducted and presented as a bubble chart and cnetplot, respectively.

Given that the aberrant *PSMA7* expression possibly adversely influenced the clinical outcome of various cancers (Fig. 4B, C, Supplementary Fig. S5A–E), we then explored the possible molecular mechanism linking high expression of *PSMA7* to tumorigenesis. With this regard, the dependency of tumor cells on *PSMA7* was evaluated by the genome-scale CRISPR-Cas9 knockout screens in the 1095 cancer cell lines extracted from the Depmap^48^ database. The *PSMA7* depletion resulted in significantly negative screen scores (concentrate range: − 2.0 to − 3.0) in the majority of types of cancers (Fig. 5A), highlighting that *PSMA7* was required for tumor cell growth as previously reported^23,31,32^.

Furthermore, via the STRING tool, the specific interaction network between 50 *PSMA7*-binding proteins supported by experimental evidence was visualized in Fig. 5B. In parallel with this, the top 100 genes with strong correlation with *PSMA7* expression were generated by the GEPIA2 online tool. Notably, *PSMA7* expression was positively correlated with selected 18 genes (all P ≤ 0.001) (details in Fig. 5C). In good accordance with this, validation by TIMER2 revealed the positive link of *PSMA7* to each of the aforesaid 18 genes across most TCGA cancers (Fig. 5D). By overlapping the above two groups via the Jvenn^49^, nine common genes were identified, including *ADRM1* (*ADRM1* 26S Proteasome Ubiquitin Receptor), *POMP* (Proteasome Maturation Protein) , *PSMA2* (Proteasome 20S Subunit Alpha 2), *PSMA4* (Proteasome 20S Subunit Alpha 4), *PSMA5* (Proteasome 20S Subunit Alpha 5), *PSMB1* (Proteasome 20S Subunit Beta 1) , *PSMB2* (Proteasome 20S Subunit Beta 2), *PSMD7* (Proteasome 26S Subunit, Non-ATPase 7) and *PSMD14* (Proteasome 26S Subunit, Non-ATPase 14 ) (Fig. 5E).

Next, we subjected those *PSMA7*-interacting proteins and genes whose expression harbored connections with *PSMA7* to the enrichment analysis. As foreseen, the KEGG-based findings manifested that the proteasome, cell cycle, ubiquitin-mediated proteolysis, and nucleocytoplasmic transport might participate in *PSMA7-*mediated tumorigenesis (Fig. 5F). Besides, GO data revealed that most genes were concentrated on protease activity-related pathways and cellular processes, especially proteasome binding or complex, threonine-type peptidase and endopeptidase activity, endopeptidase activity or complex, and peptidase complex, to guide the biological processes such as anaphase—promoting complex—dependent catabolism, RNA polymerase II transcriptional regulation, and control of cell cycle progression (Fig. 5G, Supplementary Fig. S9A, B). These results documented that aberrant *PSMA7* expression greatly influenced UPS status and thus mediated various downstream biological processes, eventually promoting tumor cell growth.

### Candidate *PSMA7*-resistant and sensitive compounds

**Figure 6:**
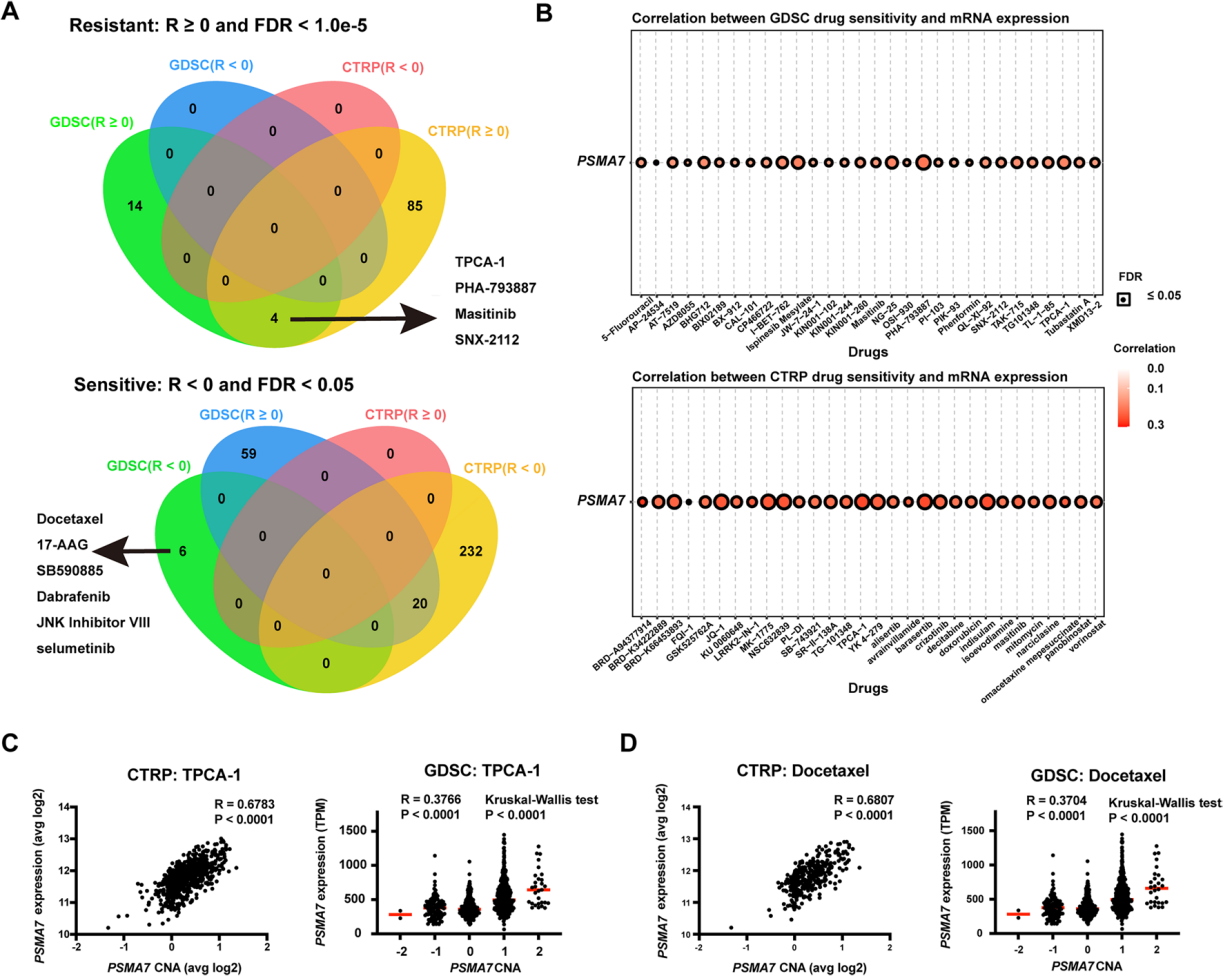
Candidate *PSMA7-*resistant and sensitive compounds for TCGA cancers. The GSCA tool includes genomic data of diverse TCGA cancers and compounds from the genomics of drug sensitivity in cancer (GDSC) and cancer therapeutics response Portal (CTRP) databases. Using GSCA, we screened the potential agent that strongly correlated with *PSMA7* expression. (**A**) An intersection analysis via Jvenn was performed to find the potential *PSMA7*-resistant and sensitive compounds in GDSC and CTRP. (**B**) The top 30 ranked agents that positively correlated with *PSMA7* expression in GDSC and CTRP were also supplied, respectively. Based on the data from both drug databases, the relationship of *PSMA7* expression with amplification was evaluated in cancer cell lines treated with (**C**) TPCA-1 and (**D**) Docetaxel, respectively.

Elevated *PSMA7* expression was considered a prognostic molecular biomarker that might become a new treated target for different types of tumors. However, previous studies suggested the potential involvement of increased PSMA7 protein levels in drug resistance. To uncover the drug-response landscape of cancers expressing upregulated *PSMA7*, we analyzed the potential connection of drug sensitivity with this gene mRNA expression across pan-cancers based on the drug sensitivity in cancer (GDSC) and cancer therapeutics response portal (CTRP) databases. Overall, positive correlation (R) values with FDR ≤ 1.0e−5 were obtained in compounds of 89 (of 481) and 18 (of 251) in CTRP and GDSC, respectively, which were considered *PSMA7*-resistant agents (Fig. 6A). Figure 6B displays the top 30 ranked resistant compounds in each drug library. To further find the candidate most *PSMA7*-resistant agents, we overlapped the data and identified four shared compounds in both CTRP and GDSC (Fig. 6A**, **Table 2). Since *PSMA7* overexpression was validated due to amplification in clinical samples with different types of cancers, we further asked whether *PSMA7* amplification showed poor response to these resistant agents, for instance, the first-ranking compound TPCA-1^50^. As expected, strong positive correlations existed between *PSMA7* expression and amplification in cell lines with TPCA-1 treatment based on the data from GDSC and CTRP^51^ (Fig. 6C). Using the genomic data from the catalogue of somatic mutations in cancer (COSMIC)^52^ database, we found that COSMIC-annotated *PSMA7* copy number gain and overexpression occurred in a total of nine cancer cell lines treated with TPCA-1 in GDSC and CTRP (Supplementary Tab. S14). Besides, almost all of these cell lines had high half maximal inhibitory concentrations (IC50) and poor area-under-the-curve (AUC) sensitivity scores for TPCA-1, suggesting that amplification of *PSMA7* might protect cancer cell lines from TPCA-1 treatment. These results manifested that *PSMA7*-related cancers might not benefit from these potentially resistant agents, which required much more validation in future research.

**Table 2:** Candidate *PSMA7*-resistant and sensitive compounds in GDSC and CTRP databases.

Compounds	Ranking-GDSC	R-GDSC	Ranking-CTRP	R-CTRP	R-GDSC + R-CTRP	Ranking
Resistant: R ≥ 0 and FDR < 1.0e−5						
TPCA-1	3	0.18775856	2	0.260356754	0.448115314	1
PHA-793887	1	0.206831292	42	0.203181836	0.410013128	2
Masitinib	13	0.161537014	17	0.227204315	0.388741329	3
SNX-2112	12	0.161659721	77	0.177990594	0.339650315	4
Sensitive: R < 0 and FDR < 0.05						
Docetaxel	1	− 0.118107832	/	/	/	/
17-AAG	2	− 0.114885903	/	/	/	/
SB590885	3	− 0.106534666	/	/	/	/
Dabrafenib	4	− 0.09261363	/	/	/	/
JNK Inhibitor VIII	5	− 0.083405322	/	/	/	/
Selumetinib	6	− 0.075319267	/	/	/	/

GDSC, genomics drug sensitivity in cancer; CTRP, cancer therapeutics response portal; R, correlation coefficient; FDR, false discovery rate; JNK, c-Jun N-terminal kinase.

Although *PSMA7*-resistant compounds accounted for the majority, we found six compounds with negative R-values and FDR ≤ 0.05 in GDSC that might be sensitive to cancers with elevated *PSMA7* expression (Fig. 6A, Table 2). The first-ranking agent was Docetaxel, an efficient microtubule depolymerization inhibitor^53^ which had been approved to treat multiple types of tumors such as advanced breast cancer^54^, gastric cancer^55^, and melanoma^56^. Consistent with the aforementioned finding, *PSMA7* expression in the cell lines treated with Docetaxel was also positively correlated with amplification in both GDSC and CTRP (Fig. 6D). Furthermore, we observed that most of the above nine cell lines with *PSMA7* copy number gain and elevated expression achieved low IC50 to Docetaxel treatment (Supplementary Tab. S14), highlighting the promising therapeutic value of this agent for *PSMA7*-related cancers. The data above generally described the drug-response pattern of abnormal *PSMA7* in cancer cell lines that need further exploration in future work.

## Discussion

CNA is a common phenomenon regulating target gene expression in a wide variety of cancers^1,2^. Recent advances manifested that 20q was widely amplified and gained in solid tumors^3,12^. Most of the 20q CNAs and some potential cancer-driver genes conferred poor prognosis and therapeutic vulnerability by influencing immortalization, genomic instability, apoptosis, and proliferation^57^. In myeloid hematological malignancies, however, deletion or loss of 20q and the involved common-deletion region (CDR) candidate genes correlated with a relatively good prognosis as a de novo chromosomal event^58^. These findings emphasized the heterogeneous role of 20q CNAs in human cancers. In this study, we for the first time revealed that 20q13.33 was the most frequently amplified genomic loci at a pan-cancer level. Notably, aberrant high expression of the *PSMA7* gene at 20q13.33 due to amplification conferred poor prognosis and therapeutic vulnerability in various cancers^12,25^. In this regard, by querying GDSC and CTRP databases, we proposed several candidate compounds, holding the promise to develop new therapeutics for relevant cancers.

In the past years, the correlation between elevated *PSMA7* expression with adverse prognosis was reported by sporadic research^12,21–28^. Recently, a TCGA-based pan-cancer study mentioned briefly the prognostic valus of 49 proteasome family member genes including *PSMA7*^59^, which indeed serves as a significant precursor to our own research. During the explorations of CNA of chr20 using similar genomic data from TCGA tumors, we identified *PSMA7* as the most potential target at the newly found amplicon of 20q13.33. Then we at a pan-cancer level systematically explored the status of amplification and expression of the *PSMA7* gene and their clinical significances in terms of prognosis and potential therapeutic opportunity. Distinguishing from the previous pan-cancer research focusing on the prognostic values of *PSMA7 *^59^, we further investigated the underlying mechanism linking high *PSMA7* expression to poor prognosis. Analysis of proteins interacting or genes correlating with *PSMA7* revealed broad functions of *PSMA7* in multiple cellular processes^13^, especially networks with proteasome binding or complex. Moreover, we found that *PSMA7* was significantly essential for tumor cell viability in the CRISPR-mediated loss of function screens of 1095 cancer cell lines. These findings suggested that the PSMA7-mediated proteasome activity might necessarily support tumor cell survival, as reported previously^23,31,32^. Moreover, Hochstrasser et al. found that elevated cellular PSMA7 levels increased preferentially the assembly of PSMA7-PSMA7 proteasome alternative isoform and protected tumor cells from the environmental pressure induced by heavy metals^20^. However, more experiment-based evidence is warranted to prove this hypothesis.

Notably, we discovered that *PSMA7* expression displayed a positive link to TMB and MSI in diverse tumors, figuring out the potential role of *PSMA7* in DNA damage repair for tumor cells^45^. According to the two-hit hypothesis of tumorigenesis^60^, amplification of 20q13.33 might serve as the first “hit”, and aberrant high expression of *PSMA7* might contribute to the second “hit” required for overt tumorigenesis by introducing more genetic lesions. Interestingly, *PSMA7* can be used as a plausible immunotherapy predictive biomarker, given its correlation with TMB^47^. Besides, we further identified a cohort of other differentially overexpressed genes between samples with and without *PSMA7* amplification, revealing a co-occurrence amplification pattern of *PSMA7* and other genes across whole chromosomes, especially 20q. One noteworthy co-amplified gene was *LAMA5* located at 20q13.33, a member of the laminin family that encoded the α chain of the extracellular matrix protein laminin 5^61^. This gene was found to promote migrations of some tumors^62,63^ and thus achieved a comparable score with *PSMA7* in cancer in the current study (Fig. 1D). The amplification of *LAMA5* which harbored the highest gene expression than any other CNA types was indeed observed in 23 (of 32) types of TCGA tumors in cBioPortal (Supplementary Fig. S10 A, B). However, genome-scale CRISPR-Cas9 knockout screens showed that *LAMA5* depletion resulted in nearly zero in almost all types of cancers, suggesting *LAMA5* was not required for tumor cell survival (Supplementary Fig. S10C). Therefore, we inferred that the chromosome amplicon 20q13.33 might be the work unit in which *PSMA7* but at least not *LAMA5* functioned as a cancer-promoting factor. Given its critical role in tumorigenesis, therapeutic compounds targeting the *PSMA7* pathway were screened using CTRP and GDSC databases, with six of them showing therapeutic potential. Further validation to test the anti-tumor effects of these candidate compounds will provide more evidence to support this.

Taken together, our study supported *PSMA7* as the crucial gene at the 20q amplification hotspot and a potential new biomarker for the diagnostic testing, prognostic evaluation, and molecular therapeutic development of human cancers. Identification of several chromosome 20q amplicons, especially the novel amplicon 20q13.33 consisting of the amplified *PSMA7* and other overexpressed genes, presents a more comprehensive landscape of genomic amplification of 20q, and the oncogenic role and underlying molecular mechanism of *PSMA7* at a pan-cancer level.

## Methods

### Genetic variation analysis

The GISTIC2.0 pipeline^34–36^ was applied to analyze the copy-number raw data from 33 types of TCGA tumors and achieve the total segmented copy-number profile and the broad and focal CNA regions across the whole genome. The results were visualized as a heatmap and detailed chromosome frequency plots. The online tool GISTIC2 from Broad Firehose^35^ was employed to obtain statistical data for each chromosome arm and detailed amplification frequency plots across the whole genome in 30 types of TCGA tumors. The “TCGA Pan Cancer Atlas Studies” of the “Quick select” module of the cBioPortal approach^37,38^ was operated with “Explore Selected Studies” to download the data for "CNA genes" from the “Summary” module. We summarized the amplification frequency of each chr20 subband and performed a Mann–Whitney U test in which a P value less than 0.05 was considered statistically significant. The enrichment analysis of genes at the chromosome subband with the highest amplification frequency was performed by the KEGG and GO. Additionally, we employed another tool Phenolyzer^39^ to explore the potential link of genes to cancer.

For *PSMA7*, the “Cancer Types Summary” section on this web was queried to quickly acquire the genomic variations including frequency, mutation type, and CNAs of this gene in TCGA tumors. The *PSMA7* expression level in various types of putative CNA was investigated in the “PLOT” module. Besides, the “Genomic Alterations” module in the “Comparison/Survival” section was employed to get the difference in co-occurrence pattern between other altered genes and amplified *PSMA7* in TCGA patients with or without *PSMA7* copy number amplification*.* We focused on the differential genes with statistically significant alteration frequencies above the 5.5 log ratio and observed their chromosomal and subband distributions.

According to the GSCA database^40^, the “Copy Number Alteration (CNA)” module in the “Mutation” section was employed to reveal the genetic alteration of *PSMA7* in TCGA tumors. The CNA frequency, genotype status, and their association with survival prognosis were observed in the “CNA summary”, and “CNA & Survival” modules, respectively. The log-rank and Cox regression tests were each applied for the prognosis estimation of CNA on OS, PFS, DFI, and DSS. The multivariate Cox regression analysis was further performed to evaluate whether the amplification of *PSMA7* represented an independent factor by adjusting established clinical and molecular variables. The relationship of *PSMA7* CNA with gene expression was evaluated in the “CNA & Expression” module. In addition, the “Pan-Cancer” section of the Assistant for Clinical Bioinformatics web was employed to estimate the possible link of *PSMA7* expression to TMB and MSI.

### Differentially expressed gene analysis

The *PSMA7* expression across TCGA tumors was compared with that in normal samples through the “Gene_DE” section of the TIMER2 online^41^. Notably, for tumors with rare numbers of normal samples, the adjacent healthy volunteer samples of the GTEx dataset were added. Then *PSMA7* expression in those tumors was reevaluated by the “Expression Analysis-Box Plots” section of the GEPIA2^42^. Furthermore, the “Pathological Stage Plot” section was operated with *PSMA7* to investigate gene expression at each pathological stage of diverse TCGA cancers. We presented these results in violin plots.

### Survival analysis

With the setting of the median expression level of *PSMA7* as the cutoff point, we operated the GEPIA2 to reveal the clinical outcome effect of gene expression on OS and DFS across TCGA cancers. The data were generated by the “Survival Map” and “Survival Analysis” modules and displayed as survival maps and log-rank survival plots, respectively. In parallel, according to the K-M plotter tool^44^, the prognosis implication of *PSMA7* expression was also estimated using the log-rank test via the “mRNA genechip” module for breast, ovarian, lung, and gastric cancers (Affy ID for 201114_x_at), as well as the “mRNA RNA-seq” module for liver cancer (RNAseq ID for 5688), across the GEO cohorts. The “Auto select best cutoff” was regarded as the cutoff point. The survival indexes included OS, distant metastasis-free survival (DMFS), PFS, FP, RFS, PPS, and DSS. P values below 0.05 were recognized with statistical differences.

### CRISPR-Cas9-mediated *PSMA7* loss of function analysis

The Depmap database^48^ captured the data of the whole genome CRISPR knockout screens in exceeding 1000 cancer cell lines. With inputting the gene “*PSMA7*″, the “Perturbation Effects” module displayed the influence of *PSMA7* depletion on cancer cell viability using the dataset of "CRISPR (DepMap Public 23Q2 + Score, Chronos)”. The data for the DepMap CRISPR dependency scores were visualized as box plots. A lower score indicates a higher likelihood that *PSMA7* is essential in a given cell line. A score of 0 and -1 represents that *PSMA7* is dispensable or comparable to the median of all pan-essential genes, respectively.

### Enrichment analysis of *PSMA7*-related genes

By inputting “PSMA7”, we identified the PSMA7-interacting proteins with experimental evidence in the organism “Homo sapiens” via STRING online. The major filtered conditions were listed in the following: the definition of network boundaries limited by “evidence”, active interaction sources originated from “experiments”, “Low confidence (0.150)” selected for the smallest required interplay score, and “no more than 50 interactors” in 1^st^ shell for showing maximum interactors.

Besides, the first 100 genes whose expressions were linked to *PSMA7* were acquired in the “Similar Gene Detection” section of GEPIA2 by combining data from TCGA tumors and healthy volunteer samples. We then explored the potential link of *PSMA7* expression to these chosen genes through the Pearson correlation test. The data were shown as dot plots with log2 TPM. In addition, via the Spearman rank correlation test, we further validated the association of these selected genes with *PSMA7* expression in the “Gene_Corr” section of TIMER2. Finally, we interpreted the result into a heat diagram.

Moreover, we overlapped the aforementioned two gene sets by Jvenn^49^ to obtain the intersection genes. Subsequently, the union genes were subjected to the KEGG and GO enrichment analysis. Briefly, all genes were functionally annotated by DAVID (the database for annotation, visualization, and integrated discovery), under the filtered conditions of chosen identifier “OFFICIAL_GENE_SYMBOL” and species “Homo sapiens”. The final results for the KEGG enriched pathway and GO were presented as bubble charts and cnetplots with R languages, respectively. P values below 0.05 were recognized with statistical differences.

### Potential *PSMA7-*resistant and sensitive drug screening

The GSCA web^40^ integrates the tumor data from diverse TCGA cancers and compounds exceeding 750 from the GDSC and CTRP databases. The “Drug” module was applied to reveal the relationship of *PSMA7* expression with the IC50 representing the drug susceptibility. For resistant (R-value: positive) and sensitive (R-value: negative) agent analysis, the FDR values below 1.0e-5 and 0.05 were considered statistically significant in each. Intersection analysis via the Jvenn online tool was conducted to obtain the candidate *PSMA7*-resistant and sensitive compounds in GDSC and CTRP. The common potential agents were re-ranked by the sum of the R-value in both drug libraries. Additionally, the top 30 ranked compounds positively correlated with *PSMA7* expression were visualized in GDSC and CTRP, respectively. The copy number data of cancer cell lines from COSMIC (Version 99, GRCh38)^52^ was applied to evaluate the potential association of *PSMA7* CNAs with the sensitivity of specific compounds in GDSC and CTRP. Finally, the gene expression and copy number profile data from both drug libraries^51^ were used to analyze the possible relationship between *PSMA7* expression and CNAs in cancer cell lines under detailed agent treatment by the correlation analysis and Kruslal-Wallis test.

### Supplementary Information

Supplementary Information.

## Data Availability

The data produced during the current research are attached in the issued main text and the additional files. All bioinformatics tools mentioned during the analysis process are public to access. Please contact shengesther@163.com if someone wants to request the data from this study.
