# Supplementary material for: Pan-caner analysis identifies PSMA7 as a targets for amplification at 20q13.33 in tumorigenesis
Source: Sci Rep. 2024 Feb 6;14:3034. doi: 10.1038/s41598-024-53585-0 (PMC10847487; doi:10.1038/s41598-024-53585-0)
Supplement: Supplementary file 1 — Supplementary Information. [file 41598_2024_53585_MOESM1_ESM.docx]

**Supplementary information**

**Supplemental Tables**

**Table S1. Broad CNAs in each chromosome arm across TCGA cancers identified by GISTIC2.0.**

| **Arm** | **# Genes** | **Amp. frequency** | **Amp. frequency score** | **Amp. z-score** | **Amp. q-value** | **Del.**  **frequency** | **Del. frequency score** | **Del. z-score** | **Del. q-value** |
| --- | --- | --- | --- | --- | --- | --- | --- | --- | --- |
| 1p | 3092 | 0.15 | 0.18 | -0.459 | 1 | 0.16 | 0.19 | 3.25 | **0.00109** |
| 1q | 2863 | 0.34 | 0.36 | 44.9 | **0** | 0.07 | 0.1 | -17.1 | 1 |
| 2p | 1500 | 0.18 | 0.19 | -4.83 | 1 | 0.08 | 0.1 | -26.3 | 1 |
| 2q | 2495 | 0.14 | 0.15 | -9.47 | 1 | 0.09 | 0.11 | -19.7 | 1 |
| 3p | 1531 | 0.13 | 0.17 | -8.44 | 1 | 0.25 | 0.29 | 17.8 | **0** |
| 3q | 1708 | 0.26 | 0.29 | 19.5 | **0** | 0.11 | 0.15 | -13.2 | 1 |
| 4p | 693 | 0.07 | 0.1 | -27.1 | 1 | 0.28 | 0.3 | 15.7 | **0** |
| 4q | 1467 | 0.05 | 0.07 | -29.5 | 1 | 0.28 | 0.3 | 20.2 | **0** |
| 5p | 456 | 0.28 | 0.31 | 16.7 | **0** | 0.1 | 0.14 | -18.9 | 1 |
| 5q | 2185 | 0.15 | 0.19 | -2.26 | 1 | 0.21 | 0.24 | 10.4 | **0** |
| 6p | 1664 | 0.17 | 0.2 | -1.96 | 1 | 0.15 | 0.18 | -5.96 | 1 |
| 6q | 1304 | 0.11 | 0.14 | -15.5 | 1 | 0.23 | 0.26 | 10.5 | **0** |
| 7p | 926 | 0.37 | 0.4 | 40.6 | **0** | 0.06 | 0.09 | -25.5 | 1 |
| 7q | 1717 | 0.34 | 0.37 | 39 | **0** | 0.07 | 0.1 | -21 | 1 |
| 8p | 795 | 0.2 | 0.27 | 9.71 | **0** | 0.28 | 0.35 | 27.3 | **0** |
| 8q | 1249 | 0.39 | 0.41 | 46.6 | **0** | 0.07 | 0.11 | -20.6 | 1 |
| 9p | 621 | 0.11 | 0.16 | -14 | 1 | 0.32 | 0.36 | 29.3 | **0** |
| 9q | 1610 | 0.11 | 0.15 | -12.3 | 1 | 0.25 | 0.29 | 17.7 | **0** |
| 10p | 767 | 0.14 | 0.18 | -9.63 | 1 | 0.23 | 0.27 | 9.17 | **0** |
| 10q | 1968 | 0.08 | 0.11 | -19.7 | 1 | 0.27 | 0.29 | 21.4 | **0** |
| 11p | 1162 | 0.09 | 0.12 | -21.1 | 1 | 0.22 | 0.24 | 5.82 | **6.01e-09** |
| 11q | 2133 | 0.1 | 0.14 | -14.2 | 1 | 0.22 | 0.25 | 12.5 | **0** |
| 12p | 804 | 0.23 | 0.26 | 8.1 | **9.10e-16** | 0.12 | 0.15 | -16 | 1 |
| 12q | 2055 | 0.18 | 0.2 | -0.464 | 1 | 0.11 | 0.14 | -14.8 | 1 |
| 13q | 1092 | 0.13 | 0.18 | -7.47 | 1 | 0.3 | 0.34 | 27.4 | **0** |
| 14q | 1829 | 0.11 | 0.15 | -12.3 | 1 | 0.24 | 0.27 | 15.2 | **0** |
| 15q | 2082 | 0.08 | 0.11 | -20 | 1 | 0.23 | 0.25 | 12.3 | **0** |
| 16p | 1378 | 0.18 | 0.22 | 1.6 | **0.173** | 0.17 | 0.2 | -2.42 | 1 |
| 16q | 1070 | 0.13 | 0.18 | -8.77 | 1 | 0.26 | 0.3 | 18.5 | **0** |
| 17p | 937 | 0.1 | 0.15 | -14.3 | 1 | 0.35 | 0.39 | 37.8 | **0** |
| 17q | 2298 | 0.19 | 0.23 | 7.15 | **1.60e-12** | 0.15 | 0.19 | -2.23 | 1 |
| 18p | 212 | 0.14 | 0.19 | -10.8 | 1 | 0.25 | 0.29 | 10.4 | **0** |
| 18q | 644 | 0.09 | 0.14 | -18.8 | 1 | 0.31 | 0.34 | 26.1 | **0** |
| 19p | 1331 | 0.16 | 0.19 | -5.73 | 1 | 0.17 | 0.2 | -2.83 | 1 |
| 19q | 2402 | 0.19 | 0.22 | 6.28 | **5.62e-10** | 0.15 | 0.19 | -1.59 | 1 |
| 20p | 583 | 0.33 | 0.36 | 30.1 | **0** | 0.08 | 0.11 | -22.9 | 1 |
| 20q | 1092 | 0.37 | 0.39 | 40.5 | **0** | 0.04 | 0.06 | -31 | 1 |
| 21q | 750 | 0.12 | 0.15 | -15.5 | 1 | 0.22 | 0.25 | 5.81 | **6.27e-09** |
| 22q | 1258 | 0.1 | 0.15 | -14.7 | 1 | 0.3 | 0.33 | 26.3 | **0** |
| Xp | 945 | 0.11 | 0.14 | -18.8 | 1 | 0.2 | 0.23 | 1.51 | **0.116** |
| Xq | 1533 | 0.13 | 0.16 | -12.6 | 1 | 0.17 | 0.2 | -3.25 | 1 |

CNAs, copy number alterations; TCGA, the cancer genome atlas ; GISTIC, Genomic Identification of Significant Targets in Cancer; Amp., amplification; Del., deletion; P value less than 0.25 is shown in bold.

**Table S2. Focal amplifications and involved genes in the whole chromosome across TCGA cancers identified by GISTIC2.0.**

| **Cytoband** | **Q value** | **Residual q value** | **Wide peak boundaries** | **Genes in wide peak** |
| --- | --- | --- | --- | --- |
| 7p11.2 | 0 | 0 | chr7:54995666-55026261 | *EGFR* |
| 11q13.3 | 0 | 0 | chr11:69649951-69666620 | *CCND1, ORAOV1* |
| 8q24.21 | 0 | 1.4465e-312 | chr8:127727004-127754638 | *MYC, CASC11* |
| 3q26.2 | 2.98e-249 | 2.98e-249 | chr3:169685704-169791126 | *TERC, MYNN, ACTRT3* |
| 19q12 | 1.07e-224 | 5.11e-180 | chr19:29815851-29825968 | *CCNE1* |
| 17q12 | 2.75e-248 | 3.45e-176 | chr17:39697302-39720408 | *ERBB2* |
| 12q15 | 3.84e-204 | 4.08e-143 | chr12:68817444-68866975 | *CPM, MDM2* |
| 1q21.3 | 3.15e-177 | 3.62e-142 | chr1:150598510-150679701 | *ENSA, GOLPH3L* |
| 8p11.23 | 4.24e-173 | 3.71e-114 | chr8:38313004-38379371 | *WHSC1L1* |
| 5p15.33 | 4.20e-64 | 4.20e-64 | chr5:1287905-1299909 | *TERT* |
| 4q12 | 6.09e-54 | 9.38e-42 | chr4:54292185-54293359 | *PDGFRA* |
| 15q26.3 | 2.31e-37 | 2.31e-37 | chr15:98513685-99235920 | *IGF1R, SYNM, TTC23, PGPEP1L, FAM169B, MIR4714, RAIN, LUNAR1* |
| 20q13.2 | 1.20e-41 | 2.39e-37 | chr20:53540389-53810936 | *ZNF217, LOC101927770, LOC105372672* |
| 20q11.21 | 4.22e-48 | 4.08e-37 | chr20:31597009-31728219 | *BCL2L1, ID1, COX4I2, MIR3193, ABALON* |
| 12q14.1 | 1.08e-125 | 3.51e-36 | chr12:57739474-57763497 | *CDK4, CYP27B1, TSPAN31, AGAP2, MIR6759,* |
| 7q21.2 | 6.99e-34 | 9.05e-34 | chr7:92549789-92847353 | *CDK6, FAM133B, LOC101927497* |
| Xq28 | 1.37e-30 | 1.37e-30 | chrX:153870069-154010438 | *ARHGAP4, AVPR2, HCFC1, L1CAM, RENBO, NAA10, TMEM187, MIR3202-2, MIR3202-1, HCFC1-AS1, LCA10* |
| 12p12.1 | 3.35e-46 | 1.57e-30 | chr12:25186964-25217765 | *KRAS, CASC1, ETFRF1* |
| 8p11.21 | 4.53e-87 | 2.50e-30 | chr8:41871229-42077038 | *ANK1, KAT6A, LOC105379393* |
| 1p34.2 | 6.28e-30 | 6.28e-30 | chr1:39912236-39951670 | *MFSD2A* |
| 12p13.33 | 9.51e-39 | 5.21e-27 | chr12:1-871473 | *NINJ2* |
| 6p21.1 | 2.2e-35 | 5.47e-25 | chr6:43825631-43925200 | *LINC01512* |
| 6p22.3 | 7.34e-34 | 5.81e-24 | chr6:20380039-20535464 | *E2F3, CDKAL1* |
| 17q25.1 | 3.41e-41 | 2.09e-21 | chr17:75585896-75600347 | *MYO15B* |
| 14q13.3 | 5.00e-25 | 4.52e-19 | chr14:36116381-36802605 | *PAX9, NKX2-1, NKX2-8, MBIP, SLC25A21, SFTA3, NKX2-1-AS1, PTCSC3, LINC00609* |
| 13q34 | 2.73e-27 | 1.26e-18 | chr13:113104424-113659178 | *ATP4B, F7, F10, LAMP1, TFDP1, CUL4A, PROZ, TMCO3, DCUN1D2, PCID2, GRTP1, ADPRHL1, GRTP1-AS1, LOC101928841, MIR8075, F10-AS1,* |
| 19p13.12 | 2.44e-18 | 2.44e-18 | chr19:15227331-15297802 | *BRD4, EPHX3* |
| 18q11.2 | 9.19e-16 | 9.19e-16 | chr18:22773904-22932197 | *LOC101927571* |
| 1q32.1 | 1.17e-35 | 9.77e-13 | chr1:204401287-204579389 | *MDM4, PIK3C2B, PPP1R15B* |
| 9p13.3 | 3.16e-15 | 1.18e-12 | chr9:35654482-35714692 | *CA9, RMRP, TLN1, TPM2, ARHGEF39, CCDC107, MIR6852* |
| 19q13.2 | 1.38e-52 | 5.30e-12 | chr19:39279756-39395555 | *GMFG, PAF1, SAMD4B, MED29, LRFN1, IFNL1* |
| 4p16.3 | 1.61e-10 | 1.61e-10 | chr4:1777070-1815700 | *FGFR3, LETM1* |
| 11p13 | 1.88e-09 | 1.88e-09 | chr11:35222498-35237859 | *CD44* |
| 2q31.2 | 1.69e-07 | 1.69e-07 | chr2:177218536-177290110 | *NFE2L2, HNRNPA3, LOC100130691, MIR3128* |
| 10q22.2 | 2.55e-07 | 2.55e-07 | chr10:74796031-75464111 | *VDAC2, KAT6B, DUSP13, C10orf11, ZNF503, COMTD1, SAMD8, ZNF503-AS1, DUPD1, ANF503-AS2, LOC101929165, LOC101929234* |
| 6q21 | 4.33e-09 | 4.10e-07 | chr6:106736955-106842825 | *MIR587, LOC100422737* |
| 10p15.1 | 1.23e-06 | 1.23e-06 | chr10:5469907-5595978 | *CALML3, CALML5, CALML3-AS1, LOC105376382* |
| 22q11.22 | 5.06e-06 | 5.06e-06 | chr22:18131163-21785126 | *ARVCF, COMT, CRKL, GGT3P, GP1BB, GSC2, SERPIND1, PI4KA, SEPT5, MAPK1, PRODH, RANBP1, SLC7A4, SLC25A1, TBX1, CLDN5, HIRA, UBE2L3, UFD1L, ZNF74, DGCR6, LZTR1, CLTCL1, DGCR14, CDC45, P2RX6, SNAP29, DGCR2, TXNRD2, USP18, HIC2, TSSK2, SDF2L1, PPIL2, DGCR11, DGCR9, DGCR5, DGCR10, TRMT2A, POM121L8P, YPEL1, ZDHHC8, MED15, TUBA8, DGCR8, GNB1L, MRPL40, RTN4R, C22orf29, THAP7, TMEM191A, KLHL22, DGCR6L, RIMBP3, SCARF2, TUBA3FP, C22orf39, TANGO2, LINC00895, LINC00896, AIFM3, RIMBP3C, YDJC,CCDC116, POM121L4P, LOC284865, PI4KAP2, CCDC188, LRRC74B, BCRP2, MIR130B, MIR185, THAP7-AS1, P2RX6P, RIMBP3B, FAM230B, LOC642643, TMEM191C, FAM230A, MIR649, TMEM191B, PI4KAP1, LOC729461, MIR301B, MIR1286, MIR1306, MIR3618, SEPT5-GP1BB, MIR4761, LINC01311, LOC100996335, LOC100996415, LOC100996432, LOC101927859, LOC101928891, MIR6816, LOC102725072* |
| 8q22.3 | 3.23e-67 | 2.33e-05 | chr8:101012677-101523943 | *ZNF706, GRHL2, NACAP1, FLJ42969* |
| 6q12 | 2.45e-07 | 6.08e-05 | chr6:63422856-63634954 | *PTP4A1* |
| 4q13.3 | 1.58e-12 | 9.02e-05 | chr4:73026719-73296173 | *ANKRD17, COX18* |
| 1q42.3 | 4.63e-28 | 0.0016602 | chr1:234796886-235079949 | *LINC01348, LOC101927851* |
| 14q32.33 | 6.73e-06 | 0.002379 | chr14:104705121-104867411 | *AKT1, SIVA1, INF2, ADSSL1, LINC00638, CEP170B, ZBTB42* |
| 3p25.2 | 0.0034439 | 0.0034439 | chr3:12204399-12464665 | *PPARG* |
| 11q14.1 | 5.63e-102 | 0.0052117 | chr11:77720196-78248612 | *NDUFC2, T HRSP, GAB2, AAMDC, RSF1, USP35, KCTD14, ALG8, INTS4, KCTD21, KCTD21-AS1, NDUFC2-KCTD14* |
| 13q22.1 | 4.43e-08 | 0.0061933 | chr13:73329535-73424514 | *LINC00392* |
| 5q35.3 | 0.0068048 | 0.0068048 | chr5:177033929-177471617 | *DBN1, F12,FGFR4, GRK6, SLC34A1, RGS14, LMAN2, ZNF346, PRELID1, RAB24, NSD1, PRR7, MXD3, PRR7-AS1, PFN3* |
| 9p24.1 | 0.00053416 | 0.010425 | chr9:5037462-5047230 | *JAK2* |
| 2p15 | 0.048573 | 0.048573 | chr2:60175884-62763298 | *PEX13, REL, XPO1, USP34, CCT4, B3GNT2, EHBP1, BCL11A, PAPOLG, FAM161A, KIAA1841, AHSA2,COMMD1, PUS10, TMEM17, LOC339803* |
| 7p22.3 | 6.58e-05 | 0.088425 | chr7:1-1204094 | *GPER1, PDGFA, PRKAR1B, ADAP1, SUN1, GET4, CYP2W1, DNAAF5, FAM20C, C7orf50, ZFAND2A, COX19, GPR146, HRAT92, LOC442497, MIR339, WI2-2373I1.2, LOC100507642, LOC101926963, LOC101927000, LOC101927021, LOC102723672, LOC105375115* |
| 11q22.2 | 5.20e-24 | 0.08899 | chr11:102402364-102648972 | *MMP7, MMP20, TMEM123, LOC101928424, LOC102723838* |
| 17q11.2 | 1.02e-26 | 0.11543 | chr17:28823782-29052102 | *FLOT2, ERAL1, PIPOX, FAM222B, PHF12, SEZ6, DHRS13, MIR144, MIR451A, MIR415B, MIR4732, LOC101927018* |
| 9q34.3 | 0.16004 | 0.16004 | chr9:137067020-137325415 | *GRIN1, SSNA1, TUBB4B, MAN1B1, NELFB, NDOR1, ANAPC2, DPP7, TOR4A, EXD3, SAPCD2, UAP1L1, TMEM203, SLC34A3, TPRN, CYSRT1, LRRC26, FAM166A, STPG3, NRARP, RNF224, RNF208, STPG3-AS1, MAN1B1-AS1, MIR3621, TMEM210* |
| 17p11.2 | 0.050294 | 0.43573 | chr17:20705445-21283047 | *TMEM11, USP22, DHRS7B, NATD1, CCDC144NL, LOC339260, CCDC144NL-AS1, LOC100287072, LINC01563* |

TCGA, the cancer genome atlas ; GISTIC, Genomic Identification of Significant Targets in Cancer.

**Table S3. Focal deletions and involved genes in the whole chromosome across TCGA cancers identified by GISTIC2.0.**

| **Cytoband** | **Q value** | **Residual q value** | **Wide peak boundaries** | **Genes in wide peak** |
| --- | --- | --- | --- | --- |
| 9p21.3 | 0 | 0 | chr9:21186157-23507724 | *CDKN2A, CDKN2B, IFNA1, IFNA2, IFNA4, IFNA5, IFNA6, IFNA7, IFNA8, IFNA10, IFNA13, IFNA14, IFNA16, IFNA17, IFNA22P, MTAP, CDKN2A-AS1, KLHL9, DMRTA1, IFNE, MIR31, LINC01239, MIR31HG, CDKN2B-AS1* |
| 5q11.2 | 0 | 1.9287e-312 | chr5:58964471-60492158 | *PDE4D, PART1, MIR582* |
| 10q23.31 | 3.9774e-311 | 4.41e-272 | chr10:87855381-88274281 | *PTEN, KLLN* |
| 13q14.2 | 3.10e-300 | 1.88e-264 | chr13:48301193-48490671 | *RB1, LPAR6* |
| 19p13.3 | 3.89e-259 | 3.89e-259 | chr19:1103716-1271529 | *ATP5D, GPX4, STK11, SBNO2, MIDN, CIRBP-AS1, CBARP* |
| 16q23.1 | 1.31e-264 | 1.85e-229 | chr16:78095161-79593873 | *WWOX* |
| 8p23.2 | 1.27e-234 | 1.41e-220 | chr8:2166023-6404670 | *CSMD1, LOC101927815* |
| 4q34.3 | 8.28e-283 | 1.26e-207 | chr4:177990720-182139540 | *LINC00290* |
| 2q22.1 | 3.24e-242 | 1.09e-206 | chr2:140577169-142880269 | *MIR7157* |
| 4q22.1 | 1.16e-215 | 4.46e-187 | chr4:90227129-92262630 | *CCSER1* |
| 2q37.3 | 3.26e-226 | 3.85e-149 | chr2:241222859-242193529 | *BOK, DTYMK, HDLBP, SEPT2, PDCD1, FARP2, STK25, ATG4B, THAP4, GAL3ST2, ING5, NEU4, RTP5, LOC285095, D2HGDH, LOC728323, BOK-AS1, MIR3133, LINC01237, LOC102723927* |
| 6q26 | 1.77e-170 | 1.98e-147 | chr6:161467895-162732175 | *LOC105378098* |
| 22q13.32 | 2.14e-129 | 6.39e-120 | chr22:48272949-48780733 | *FAM19A5, LOC284933, MIR3201, MIR4535* |
| 11p15.5 | 1.68e-101 | 1.69e-101 | chr11:1-549958 | *HRAS, PSMD13, RNH1, IFITM1, IFITM3, IFITM2, PKP3, SIRT3, BET1L, SIGIRR, RIC8A, PGGHG, PTDSS2, ODF3, SCGB1C1, NLRP6, ANO9, B4GALNT4, IFITM5, LINC01001, MIR6743* |
| 1p36.23 | 5.27e-246 | 1.22e-95 | chr1:7769048-8318933 | *TNFRSF9, PER3, VAMP3, UTS2, PARK7, ERRFI1, LOC102724539* |
| 3p14.2 | 1.73e-122 | 4.93e-77 | chr3:59717096-61561656 | *FHIT, MIR548BB* |
| 11q25 | 1.13e-94 | 3.93e-75 | chr11:133844778-135086622 | *IGSF9B, NCAPD3, ACAD8, B3GAT1, THYN1, JAM3, GLB1L2, VPS26B, GLB1L3, MIR4697HG, LOC283177, LOC100128239, LOC100507548, MIR4697* |
| 1p36.11 | 3.32e-218 | 2.02e-73 | chr1:26572635-26801443 | *ARID1A, PIGV, LOC101928728* |
| 9p23 | 1.63e-174 | 8.90e-68 | chr9:8310705-10619051 | *PTPRD, PTPRD-AS1, LOC105375972* |
| Xp21.1 | 1.17e-65 | 1.17e-65 | chrX:30855306-32593242 | *DMD,FTHL17, MIR3915* |
| 20p12.1 | 1.74e-63 | 1.7e-63 | chr20:14553293-15925872 | *MACROD2-IT1,LOC613266, MACROD2-AS1* |
| 18q23 | 7.91e-86 | 2.92e-62 | chr18:77990855-80373285 | *NFATC1, CTDP1, TXNL4A, ADNP2, KCNG2, SALL3, RBFA, PQLC1, PARD6G, LOC284241, ATP9B, HSBP1L1, PARD6G-AS1, RBFADN* |
| 6p25.3 | 7.54e-55 | 7.20e-55 | chr6:1608602-2252191 | *FOXC1, GMDS* |
| 15q15.1 | 1.63e-91 | 9.49e-53 | chr15:41503703-41846257 | *TYRO3, MAPKBP1, MGA, RPAP1, MIR626, JMJD7* |
| 16p13.3 | 3.93e-47 | 1.16e-36 | chr16:5601841-7710391 | *RBFOX1, MIR8065* |
| 17q11.2 | 4.03e-41 | 1.84e-36 | chr17:30999718-31395600 | *EVI2A, EVI2B, NF1, OMG, MIR4733* |
| 10q26.3 | 1.00e-59 | 1.07e-33 | chr10:133376759-133400839 | *PAOX* |
| 7q31.1 | 4.08e-31 | 3.79e-25 | chr7:109959411-111726314 | *LRRN3, IMMP2L* |
| 18q21.2 | 4.43e-45 | 3.32e-23 | chr18:50988026-51179001 | *SMAD4* |
| 13q12.11 | 7.34e-79 | 5.12e-22 | chr13:1-19174208 | *FAM230C, ANKRD20A9P, LINC00442, LINC00408, LINC00417* |
| 17p13.1 | 1.87e-26 | 2.14e-21 | chr17:7632143-7690247 | *ATP1B2, TP53* |
| 6q16.1 | 1.17e-30 | 5.37e-21 | chr6:90585841-96563557 | *EPHA7, FUT9, UFL1, MANEA, TSG1, MIR4643, MANEA-AS1, CASC6* |
| 10p15.3 | 8.69e-18 | 8.69e-18 | chr10:1-642483 | *ZMYND11, TUBB8, MIR5699, MIR7641-2, LOC102723376* |
| 7q36.1 | 5.23e-25 | 4.42e-15 | chr7:152120330-152438989 | *KMT2C* |
| 19q13.32 | 9.64e-37 | 3.50e-14 | chr19:46741056-47779058 | *C5AR1, AP2S1, ARHGAP35, NPAS1, SLC1A5, SLC8A2, NAPA, DHX34, SAE1, KPTN, ZC3H4, CCDC9, BBC3, C5AR2, GLTSCR2, GLTSCR1, EHD2, TMEM160, MEIS3, FKRP, ZNF541, INAFM1, SNORD23, SNAR-E, MIR3191, MIR3190, NAPA-AS1, GLTSCR2-AS1* |
| 12p13.1 | 6.74e-14 | 6.60e-14 | chr12:12691300-12768198 | *CDKN1B, MIR613* |
| 1p13.2 | 1.61e-42 | 1.57e-13 | chr1:110631436-117864021 | *ADORA3, AMPD1, RHOC, ATP1A1, ATP5F1, CAPZA1, CASQ2, CD2, CD53, CD58, CHI3L2, IGSF3, KCNA3, KCND3, MOV10, NGF, NHLH2, NRAS2, OVGP1, PTGFRN, RAP1A, SLC16A1, SYCP1, TSHB, WNT2B, CSDE1, TTF2, CD101, LRIG2, TSPAN2, BCAS2, CEPT1, AP4B1, PHTF1, MAN1A2, DDX20, PTPN22, CHIA, TRIM33, RSBN1, FAM46C, ST7L, SLC22A15, LRIF1, CTTNBP2NL, FAM212B, OLFML3, TMIGD3, DCLRE1B, WDR77, VTCN1, DENND2D, SIKE1, TRIM45, VANGL1, ATP1A1-AS1, MAB21L3, DRAM2, PIFO, Corf162, SYT6, CHIAP2, DENND2C, HIPK1, AKR7A2P1, MAGI3, FAM19A3, PPM1J, C1orf137, BCL2L15, PGCP1, LOC643355, LOC643441, MIR942, LINC01160, AP4B1-AS1, MIR320B1, MIR4256, FAM212B-AS1, SLC16A1-AS1, MIR548AC, KCND3-AS1, KCND3-IT1, LOC100996251, LOC100996263, LINC01356, LOC101928718, HIPK1-AS1, LOC101928977, LOC101928995, LOC101929023, LOC101929099, LINC01525* |
| 7p22.3 | 7.03e-11 | 6.96e-11 | chr7:1-1204094 | *GPER1, PDGFRA, PRKAR1B, ADAP1, SUN1, GET4, CYP2W1, DNAAF5, FAM20C, C7orf50, ZFAND2A, COX19, GPR146, HRAT92, LOC442497, MIR339, WI2-2373I1.2, LOC100507642, LOC101926963, LOC101927000, LOC101927021, LOC102723672, LOC105375115* |
| 9q21.11 | 7.42e-30 | 3.27e-10 | chr9:38619155-68537321 | *PGM5 ,ZNF658, SPATA31A7, CNTNAP3, ANKRD20A1, FAM201A, CBWD5, LOC286297, FOXD4L3, FOXD4L4, AQP7P1, FGF7P6, SPATA31A6, GLIDR, FAM74A1, FAM74A4, ZNF658B, LOC403323, LOC440896, ANKRD20A3, ANKRD20A2, AQP7P3, PTGER4P2-CDK2AP2P2, CBWD3, LOC554249, PGM5-AS1, PGM5P2, FRG1JP, LOC642929, LINC01189, CNTNAP3P2, CBWD6, FLJ43315, SPATA31A1, FOXD4L6, FOXD4L5, FGF7P3, SPATA31A3, SPATA31A5, FAM74A3, CNTNAP3B, LOC728673, ANKRD20A4, FAM27E3, LOC100132249, FRG1HP, GXYLT1P3, FAM27C, FAM95B1, FAM27B, FAM27E2, MIR1299, MIR4477A, FAM74A7, LOC101927827, LOC101928195, LOC101928381, LOC101929583, LOC102723709, LOC102724238, LOC102724580, LOC102725126, LINC01410, LOC103908605, LOC105376064, LOC105379450, XLOC_007697* |
| 12q24.33 | 9.24e-24 | 3.50e-10 | chr12:132431227-133275309 | *GOLGA3, POLE, PXMP2, ZNF10, ZNF26, ZNF84, ZNF140, ZNF268, P2RX2, ANKLE2, CHFR, FBRSL1, PGAM5, ANHX, ZNF605, LRCOL1, ZNF891, LOC101928530, LOC101928597, MIR6763* |
| 19q13.43 | 1.18e-37 | 1.68e-09 | chr19:58068910-58617616 | *A1BG, RPS5, ZNF8, MZF1, ZNF132, UBE2M, TRIM28, ZNF274, SLC27A5, ZNF324, CHMP2A, ZNF544, ZNF446, ZSCAN18, CENPBD, ZNF329, ZBTB45, ZNF837, ZNF497, ZNF584, ZSCAN22, ZNF324B, A1BG-AS1, RNF225, MZF1-AS1, MIR4754, MIR6806, MIR6807, LOC105372483* |
| 3q13.31 | 3.50e-12 | 2.70e-09 | chr3:116714280-118508684 | *LINC00901, MIR4447* |
| 12q23.1 | 1.52e-15 | 2.90e-09 | chr12:98730223-100037494 | *ANKS1B, FAM71C, LOC101928937* |
| 21q22.3 | 4.47e-14 | 4.37e-09 | chr21:41455552-41680542 | *TMPRSS2* |
| 3p13 | 3.07e-62 | 5.30e-09 | chr3:69986161-71543355 | *FOXP1, MIR1284, SAMMSON, FOXP1-AS1* |
| 15q11.2 | 1.10e-59 | 5.67e-09 | chr15:1-25679601 | *NBEAP1, IPW, NDN, SNRPN, UBE3A, MKRN3, PWAR5, SNURF, CYFIP1, NPAP1, MAGEL2, NIPA2, SNORD107, TUBGCP5, NIPA1, PWAR1, LOC283683, GOLGA6L2, OR4N4, HERC2P3, GOLGA6L1, GOLGA8IP, SNORD108, SNORD109A, PWAR4, SNORD115-1, WHAMMP3, SNORD64, PWARSN, LINC01193, IGHV1OR15-1, GOLGA8EP, OR4M2, OR4N3P, HERC2P2, NF1P2, GOLGA6L22, CHEK2P2, LOC646214, CXADRP2, IGHV1OR15-3, REREP3, GOLGA8S,GOLGA8J, GOLGA6L6, LOC727924, GOLGA8CP, PWRN1, PWRN2, SNORD116-1, SNORD116-2, SNORD116-3, SNORD116-4, SNORD116-5, SNORD116-8, SNORD116-10, SNORD116-11, SNORD116-12, SNORD116-13, SNORD116-14, SNORD116-15, SNORD116-16, SNORD116-17, SNORD116-18, SNORD116-20, SNORD116-21, SNORD116-22, SNORD116-23, SNORD116-24, SNORD116-25, SNORD115-2, SNORD116-26, SNORD116-27, SNORD115-3, SNORD115-4, SNORD115-5, SNORD115-6, SNORD115-7, SNORD115-8, SNORD115-10, SNORD115-11, SNORD115-12, SNORD115-13, SNORD115-14, SNORD115-15, SNORD115-16, SNORD115-17, SNORD115-18, SNORD115-20, SNORD115-21, SNORD115-22, SNORD115-23, SNORD115-25, SNORD115-26, SNORD115-29, SNORD115-30, SNORD115-31, SNORD115-32, SNORD115-33, SNORD115-34, SNORD115-35, SNORD115-36, SNORD115-37, SNORD115-38, SNORD115-39, SNORD115-41, SNORD115-44, SNORD116-28, SNORD116-29, SNORD115-48, SNORD115-24, SNORD115-27, SNORD115-28, SNORD115-45, SNORD115-47, HERC2P7, MIR1268A, MIR3118-3, MIR3118-4, MIR3118-2, MIR4509-1, MIR4509-2, MIR4508, MIR5701-2, MIR5701-1, SNORD116-30, SNORD115-46, POTEB, LOC101927079, PWRN3, POTEB3, P WRN4* |
| 14q23.3 | 9.03e-16 | 8.20e-09 | chr14:66496435-67191395 | *GPHN* |
| 3p26.2 | 2.59e-13 | 1.92e-08 | chr3:1-3059102 | *CHL1, CNTN6, CNTN4, CNTN4-AS2, CHL1-AS1, LINC01266, LOC102723448* |
| 3q26.31 | 7.17e-08 | 3.44e-06 | chr3:174282016-176041981 | *NAALADL2, MIR4789, NAALADL2-AS3, NAALADL2-AS2, NAALADL2-AS1* |
| 4p16.3 | 5.14e-06 | 5.14e-06 | chr4:1-1094042 | *ATP5I, DGKQ, GAK, IDUA, MYL5, PDE6B, ZNF141, PCGF3, CPLX1, SLC26A1, FGFRL1, PIGG, ABCA11P, MFSD7, MIR571, TMEM175, ZNF595, ZNF721, ZNF718, ZNF876P, ZNF732, LOC100129917* |
| 21q11.2 | 8.75e-28 | 1.86e-05 | chr21:1-14110283 | *BAGE, CBS, CRYAA, PWP2, TPTE, U2AF1, C21orf33, ICOSLG, CYP4F29P, SMIM11A, BAGE4, BAGE3, BAGE2, LINC00313, ANKRD30BP2, SIK1, LINC00319, POTED, ANKRD20A11P, MGC39584, MIR663A, RNA5-8S5, RNA18S5, RNA28S5, TEKT4P2, MIR3118-1, MIR3156-3, MIR3687-1, MIR3648-1, LOC100507412, RNA45S5, C21orf140, MIR6724-1, MIR8069-1, LOC102723360, KCNE1B, SMIM11B, LOC102724188, LOC102724219, LOC102724354, LOC102724428, U2AF1L5, LOC102724652, LOC102724843* |
| 2p25.3 | 2.62e-05 | 2.62e-05 | chr2:1-1912857 | *ACP1, TPO, PXDN, SH3YL1, SNTG2, TMEM18, FAM150B, LINC01115, FAM110C, LOC101060385, LOC101060391, LOC105373352* |
| 1q44 | 0.00014427 | 0.00014544 | chr1:245701488-246884306 | *SCCPDH, TFB2M, SMYD3, LINC01341, CNST, LOC255654* |
| 16q24.3 | 2.90E-59 | 0.0002679 | chr16:89234015-90338345 | *AFG3L1P, GAS8-AS1, DPEP1, FANCA, GAS8, MC1R, CHMP1A, RPL13, SPG7, CDK10, VSP9D1, TUBB3, PRDM7, TCF25, CPNE7, ANKRD11, DEF8, DBNDD1, SPIRE2, CENPBD1, ZNF276, SPATA2L, SPATA33, SNORD68, VPS9D1-AS1, URAHP, LOC100287036, FAM157C, LOC101927817, LOC105371414* |
| 17q25.3 | 3.18e-06 | 0.00028231 | chr17:82740177-83257441 | *TBCD, ZNF750, B3GNTL1, METRNL, RPL23AP87* |
| 10q21.1 | 2.82e-10 | 0.00099443 | chr10:50884325-52301677 | *PRKG1, CSTF2T, MIR605, LOC102724719* |
| 17p12 | 7.63e-07 | 0.0016004 | chr17:11993313-12552764 | *MAP2K4, MIR744* |
| 9q34.3 | 0.00011407 | 0.0017455 | chr9:133736937-135626922 | *COL5A1* |
| 5q21.1 | 3.86e-38 | 0.0019742 | chr5:95858037-114363969 | *APC, CAMK4, CAST, CHD1, EFNA5, FER, LNPEP, MAN2A1, MCC, PAM, RCSK1, SRP19, ST8SIA4, REEP5, NREP, RAB9BP1, PJA2, ELL2, PPIP5K2, ERAP1, EPB41L4A-AS2, GIN1, RIOK2, EPB41L4A, ERAP2, FBXL17, YTHDC2, NUDT12, TSSK1B, TSLP, C5orf30, SLC25A46, EPB41LA4-AS1, SLCO6A1, STARD4, WDR36, DCP2, LIX1, RGMB, LINC00491, FAM174A, SLCO4C1, RGMB-AS1, TMEM232, SNORA13, MIR583, LOC100133050, LOC100289230, LOC100289673, MIR548F3, MIR548P, STARD4-AS1, LINC01023, LINC00492, NREP-AS1, LOC101927023, LOC101929710, LOC102467212, LOC102467213, LOC102467214, LOC102467216, LINC01340, CTD-2151A2.1* |
| 14q32.32 | 2.56e-09 | 0.002145 | chr14:102726033-102934699 | *TRAF3, AMN* |
| 8p21.2 | 2.23e-26 | 0.0079139 | chr8:26038931-26505510 | *BNIP3L, PPP2R2A* |
| 1p31.1 | 7.87e-15 | 0.0099705 | chr1:71234073-73311663 | *NEGR1, NEGR1-IT1, LINC01360* |
| 21q21.1 | 1.02e-10 | 0.036138 | chr21:20799951-21723675 | *NCAM2* |
| 16p13.3 | 6.43e-13 | 0.040525 | chr16:1-681770 | *ARHGDIG, HBA1, HBA2, HBM, HBQ1, HBZ, MPG, NME4, CAPN15, NPRL3, AXIN1, RGS11, RHBDL1, PIGQ, RAB11FIP3, MRPL28, DECR2, POLR3K, LUC7L, RAB40C, TMEM8A, RHBDF1, LINC00235, PDIA2, SNRNP25, FAM234A, METTL26, MCRIP2, RHOT2, WFIKKN1, PRR35, WDR90, NHLRC4, WASIR2, LOC100134368, DDX11L10, MIR3176, MIR5587, MIR6859-2, MIR6859-3, MIR6859-1, LOC105371038, LOC105371184* |
| 11q14.1 | 0.00044333 | 0.053552 | chr11:83284692-85637750 | *DLG2, TMEM126B* |
| 14q11.2 | 0.00057353 | 0.071629 | chr14:1-21155488 | *hsa-mir-1201, ANG, APEX1, PNP, RNASE1, RNASE2, RNASE3, RNASE4, RNASE6, TEP1, PARP2, EDDM3A, SLC39A2, ZNF219, OSGEP, ARHGEF40, NDRG2, CCNB11P1, EDDM3B, METTL17, OR4K5, OR11H2, OR4K1, OR4K15, RNASE7, TMEM55B, TTC5, RNASE11, TPPP2, RNASE8, OR4K14, OR4L1, OR11H6, KLHL33, LOC254028, RNASE10, OR6S1, OR4N2, OR4K2, OR4K13, OR4K17, OR4N5, OR11G2, OR11H4, RNASE9, POTEG, OR11H12, RNASE13, OR4Q3, OR4M1, RNASE12, DUXAP10, POTEM, LOC642426, LINC01296, ECRP, TMEM253, SNORD126, LOC100508046, POTEH-AS1, BMS1P17, LOC101929572, LOC101929718, MIR6717, BMS1P22* |
| 22q11.1 | 2.82e-07 | 0.071629 | chr22:1-17179733 | *IL17RA, ANKRD62P1-PARP4P3, POTEH, HSFY1P1, CECR6, CECR5, OR11H1, GAB4, CCT8L2, XKR3, TPTEP1, DUXAP8, CECR7, CECR5-AS1, POTEH-AS1, LOC100996342, BMS1P17, LOC101929350, LOC102723769, LOC102723780, LINC01297, BMS1P22* |
| Xq21.33 | 0.08554 | 0.08554 | chrX:95034000-99463613 | *DIAPH2, DIAPH2-AS1, RPA4, BRDTP1, MIR548M* |
| 8p11.21 | 0.0040933 | 0.29617 | chr8:43028712-46549322 | *FNTA, POMK, HGSNAT, POTEA* |

TCGA, the cancer genome atlas ; GISTIC, Genomic Identification of Significant Targets in Cancer.

**Table S4. Multivariate Cox regression analysis on the prognosis of TCGA-based LGG cases.**

| **Variables** | **Group** | **OS** | | **PFI** | | **DSS** | |
| --- | --- | --- | --- | --- | --- | --- | --- |
|  |  | **HR(95%CI)** | **P value** | **HR(95%CI)** | **P value** | **HR(95%CI)** | **P value** |
| **Age** | <60years |  |  |  |  |  |  |
|  | ≥60years | 4.881(2.949-7.752) | **<0.0001** | 2.279(1.514-3.352) | **<0.0001** | 4.682(2.744-7.674) | **<0.0001** |
| **Gender** | Female |  |  |  |  |  |  |
|  | Male | 1.476(0.9756-2.258) | 0.0685 | 1.008(0.7388-1.379) | 0.9619 | 1.529(0.9973-2.375) | 0.0544 |
| **Race** | White |  |  |  |  |  |  |
|  | BLACK OR AFRICAN AMERICAN | 2.019(0.8226-4.250) | 0.0887 | 0.8861(0.4263-1.647) | 0.7233 | 2.158(0.8715-4.601) | 0.066 |
|  | ASIAN | 2.933e-10(NA) | 0.9998 | 1.203(0.06728-5.678) | 0.8566 | 8.042e-12(NA) | >0.9999 |
| **Histological Type** | Astrocytoma |  |  |  |  |  |  |
|  | Oligodendroglioma | 0.7218(0.4564-1.131) | 0.1577 | 0.6511(0.4487-0.9384) | **0.0224** | 0.6568(0.4091-1.041) | 0.0765 |
|  | Oligoastrocytoma | 0.86(0.4912-1.459) | 0.5854 | 0.7409(0.4710-1.141) | 0.1826 | 0.8354(0.4644-1.449) | 0.5336 |
| **Histological Grade** | Grade 2 |  |  |  |  |  |  |
|  | Grade 3 | 2.7(1.719-4.334) | **<0.0001** | 1.432(1.023-2.016) | **0.0376** | 2.736(1.715-4.472) | **<0.0001** |
| **Tumor Status** | Tumor Free |  |  |  |  |  |  |
|  | With Tumor | 11.22(4.108-39.92) | **<0.0001** | 3.644(1.918-7.229) | **0.0001** | 4.548e11(NA) | 0.9998 |
| **Treatment Outcome First Course** | PD |  |  |  |  |  |  |
|  | SD | 0.3095(0.1913-0.4873) | **<0.0001** | 0.2425(0.1637-0.3529) | **<0.0001** | 0.29(0.1762-0.4624) | **<0.0001** |
|  | CR | 0.4632(0.1788-1.026) | 0.0808 | 0.3175(0.1604-0.5945) | **0.0006** | 0.6073(0.2305-1.327) | 0.2559 |
|  | PR | 0.1809(0.06256-0.4137) | **0.0003** | 0.2263(0.1269-0.3784) | **<0.0001** | 0.1452(0.04368-0.3572) | **0.0002** |
| **PSMA7 CNA** | Diploid |  |  |  |  |  |  |
|  | Amplification | 2.665(1.555-4.399) | **0.0002** | 1.831(1.164-2.782) | **0.0063** | 2.566(1.471-4.301) | **0.0005** |
|  | Deletion | 2.204(0.3490-7.586) | 0.29 | 1.405(0.2302-4.496) | 0.6366 | 2.288(0.3606-7.952) | 0.2701 |

TCGA, the cancer genome atlas ; LGG, brain lower-grade glioma; HR, hazard ratio; OS, overall survival; PFI, progression-free interval;

DSS, disease-specific survival; NA, not available data; PD, Progressive Disease; SD, Stable Disease; CR, Complete Remission/Response;

PR, Partial Remission/Response; CNA, copy number alteration; *P* value less than 0.05 is shown in bold.

**Table S5. Multivariate Cox regression analysis on the prognosis of TCGA-based LGG cases (including genomic alteration).**

| **Variables** | **Group** | **OS** | | **PFI** | | **DSS** | | |
| --- | --- | --- | --- | --- | --- | --- | --- | --- |
|  |  | **HR(95%CI)** | **P value** | **HR(95%CI)** | **P value** | **HR(95%CI)** | | **P value** |
| **Age** | <60years |  |  |  |  |  | |  |
|  | ≥60years | 3.611(2.131-6.036) | **<0.0001** | 1.809(1.191-2.690) | **0.0042** | 3.297(1.910-5.610) | | **<0.0001** |
| **Gender** | Female |  |  |  |  |  | |  |
|  | Male | 1.479(0.9709-2.281) | 0.0717 | 0.9905(0.7258-1.356) | 0.9522 | 1.487(0.9628-2.329) | | 0.0773 |
| **Race** | White |  |  |  |  |  | |  |
|  | BLACK OR AFRICAN AMERICAN | 1.59(0.6392-3.409) | 0.2705 | 0.8228(0.3989-1.515) | 0.5627 | 1.69(0.6761-3.656) | | 0.2164 |
|  | ASIAN | 3.028e-10(NA) | 0.9998 | 1.156(0.06452-5.498) | 0.8873 | 1.146e-11(NA) | | >0.9999 |
| **Histological Type** | Astrocytoma |  |  |  |  |  | |  |
|  | Oligodendroglioma | 0.7658(0.4266-1.338) | 0.3586 | 0.8428(0.5355-1.303) | 0.4501 | 0.6789(0.3694-1.206) | | 0.197 |
|  | Oligoastrocytoma | 1.10(0.5748-1.721) | 0.9728 | 0.7891(0.5043-1.208) | 0.2863 | 0.9667(0.5363-1.684) | | 0.9071 |
| **Histological Grade** | Grade 2 |  |  |  |  |  | |  |
|  | Grade 3 | 1.879(1.146-3.127) | **0.0135** | 1.125(0.7851-1.615) | 0.5225 | 1.84(1.097-3.136) | | **0.0224** |
| **Tumor Status** | Tumor Free |  |  |  |  |  | |  |
|  | With Tumor | 12.11(4.535-42.50) | **<0.0001** | 3.479(1.909-6.659) | **<0.0001** | | 6.169e11(NA) | 0.9998 |
| **Treatment Outcome First Course** | PD |  |  |  |  |  | |  |
|  | SD | 0.3908(0.2388-0.6233) | **0.0001** | 0.2859(0.1920-0.4184) | **<0.0001** | 0.3716(0.2234-0.5998) | | **<0.0001** |
|  | CR | 0.5022(0.1996-1.087) | 0.1067 | 0.3874(0.2025-0.7024) | **0.0028** | 0.5623(0.2084-1.262) | | 0.2031 |
|  | PR | 0.1919(0.06504-0.4510) | **0.0007** | 0.2625(0.1460-0.4432) | **<0.0001** | 0.1521(0.04483-0.3852) | | **0.0004** |
| **Subtype** | IDHwt |  |  |  |  |  | |  |
|  | IDHmut-non-codel | 0.3033(0.1664-0.5506) | **<0.0001** | 0.3706(0.2392-0.5778) | **<0.0001** | 0.2661(0.1418-0.4960) | | **<0.0001** |
|  | IDHmut-codel | 0.2617(0.1270-0.5258) | **0.0002** | 0.2598(0.1485-0.4525) | **<0.0001** | 0.2152(0.09827-0.4530) | | **<0.0001** |
| **PSMA7 CNA** | Diploid |  |  |  |  |  | |  |
|  | Amplification | 1.841(1.046-3.124) | **0.0281** | 1.1284(0.8037-1.988) | 0.2782 | 1.647(0.9199-2.840) | | 0.0814 |
|  | Deletion | 2.416(0.3805-8.464) | 0.2408 | 1.474(0.2393-4.829) | 0.5936 | 2.465(0.3861-8.734) | | 0.233 |

TCGA, the cancer genome atlas ; LGG, brain lower-grade glioma; HR, hazard ratio; OS, overall survival; PFI, progression-free interval;

DSS, disease-specific survival; NA, not available data; PD, Progressive Disease; SD, Stable Disease; CR, Complete Remission/Response;

PR, Partial Remission/Response; IDH, isocitrate dehydrogenase; WT, Wild type; CNA, copy number alteration; *P* value less than 0.05 is shown in bold.

**Table S6. Multivariate Cox regression analysis on the prognosis of TCGA-based SARC cases.**

| **Variables** | **Group** | **OS** | | **PFI** | | **DSS** | | **DFI** | |
| --- | --- | --- | --- | --- | --- | --- | --- | --- | --- |
|  |  | **HR(95%CI)** | **P value** | **HR(95%CI)** | **P value** | **HR(95%CI)** | **P value** | **HR(95%CI)** | **P value** |
| **Age** | <60years |  |  |  |  |  |  |  |  |
|  | ≥60years | 1.555(0.9540-2.579) | 0.0807 | 0.9964(0.6719-1.486) | 0.9857 | 1.421(0.8421-2.441) | 0.1936 | 1.066(0.5744-1.983) | 0.839 |
| **Gender** | Female |  |  |  |  |  |  |  |  |
|  | Male | 0.5361(0.3207-0.8914) | **0.0165** | 0.9563(0.6445-1.421) | 0.8246 | 0.4887(0.2779-0.8558) | **0.0123** | 1.157(0.6353-2.102) | 0.6314 |
| **Race** | White |  |  |  |  |  |  |  |  |
|  | BLACK OR AFRICAN AMERICAN | 1.801(0.6981-4.071) | 0.1851 | 1.547(0.6996-3.060) | 0.2408 | 1.717(0.5920-4.313) | 0.2791 | 1.391(0.4532-3.528) | 0.5201 |
|  | ASIAN | 4.452(0.2436-22.65) | 0.1526 | 1.022(0.05713-4.840) | 0.9828 | 9.794(0.5234-53.96) | **0.0327** | 1.756(0.09547-9.140) | 0.592 |
| **Histological Type** | Dedifferentiated liposarcoma |  |  |  |  |  |  |  |  |
|  | LMS | 0.7371(0.4002-1.372) | 0.3299 | 1.151(0.7028-1.899) | 0.5791 | 0.7609(0.3933-1.493) | 0.4201 | 1.211(0.3813-3.537) | 0.732 |
|  | Myxofibrosarcoma | 1.114(0.4263-2.581) | 0.8118 | 1.353(0.6283-2.714) | 0.4144 | 1.378(0.4892-3.371) | 0.5074 | 1.658(0.4734-5.216) | 0.4002 |
|  | Pleomorphic 'MFH'/ UPS | 1.901(0.8444-4.063) | 0.1059 | 1.606(0.8135-3.017) | 0.1533 | 2.006(0.7607-4.753) | 0.1304 | 9.448e-11(NA) | >0.9999 |
|  | Synovial Sarcoma-Biphasic | 4.843e-10(NA) | 0.9999 | 3.151e-10(NA) | 0.9998 | 1.486(NA) | >0.9999 | 1.102(0.2224-4.221) | 0.8938 |
|  | Synovial Sarcoma-Monophasi | 1.1658(0.3755-5.196) | 0.4354 | 1.039(0.2416-3.085) | 0.9519 | 1.21(0.1855-4.537) | 0.8055 | 1.482e-10(NA) | >0.9999 |
|  | UPS | 1.401(0.4619-3.500) | 0.504 | 2.442(1.063-5.104) | **0.0241** | 1.968(0.6438-4.994) | 0.1855 | 2.816(0.8409-8.350) | 0.0708 |
|  | Sarcoma; synovial; poorly differentiated | 58.55(2.914-396.0) | **0.0004** | 5.045e-10(NA) | 0.9999 | 2.333(NA) | >0.9999 | NA | NA |
|  | MPNST | 1.641(0.3721-5.135) | 0.4442 | 1.203(0.4336-2.859) | 0.6965 | 2.105(0.4657-6.911) | 0.2634 | 5.29e-11(NA) | >0.9999 |
|  | Desmoid Tumor | 5.955e-11(NA) | 0.9999 | 1.202(0.06613-6.003) | 0.8595 | 7.238e-13(NA) | >0.9999 | 1.776e-10(NA) | >0.9999 |
| **Tumor Status** | Tumor Free |  |  |  |  |  |  |  |  |
|  | With Tumor | 14.86(7.554-32.40) | **<0.0001** | 15.55(9.474-26.83) | **<0.0001** | 3.937e12(NA-+infinity) | 0.9998 | 25.84(12.58-58.70) | **<0.0001** |
| **PSMA7 CNA** | Diploid |  |  |  |  |  |  |  |  |
|  | Amplification | 1.741(1.058-2.873) | **0.029** | 1.371(0.9113-2.056) | 0.128 | 1.736(1.014-2.978) | **0.0441** | 1.496(0.7906-2.788) | 0.2077 |
|  | Deletion | 1.886(0.8564-3.826) | 0.0932 | 1.626(0.8795-2.828) | 0.1004 | 1.736(0.7128-3.806) | 0.1911 | 1.985(0.8505-4.256) | 0.0914 |

TCGA, the cancer genome atlas ; SARC, Sarcoma; HR, hazard ratio; OS, overall survival; PFI, progression-free interval; DSS, disease-specific survival; DFI, disease-free interval; NA, not available data; LMS, Leiomyosarcoma; UPS, Undifferentiated Pleomorphic Sarcoma; MPNST, Malignant Peripheral Nerve Sheath Tumors; CNA, copy number alteration; *P* value less than 0.05 is shown in bold.

**Table S7. Multivariate Cox regression analysis on the prognosis of TCGA-based THCA cases.**

| **Variables** | **Group** | **OS** | | **PFI** | | **DSS** | |
| --- | --- | --- | --- | --- | --- | --- | --- |
|  |  | **HR(95%CI)** | **P value** | **HR(95%CI)** | **P value** | **HR(95%CI)** | **P value** |
| **Age** | / | 1.178(1.092-1.311) | **0.0002** | 0.9927(0.9624-1.022) | 0.6351 | 1.697(NA) | **0.049** |
| **Gender** | Female |  |  |  |  |  |  |
|  | Male | 2.397(0.3908 -NA) | 0.3518 | 1.553(0.7734-2.998) | 0.1999 | 0.2816(NA) | 0.7663 |
| **Race** | White |  |  |  |  |  |  |
| **Race** | BLACK OR AFRICAN AMERICAN | 2.606(0.2622-22.33) | 0.3687 | 1.762(0.4103-5.244) | 0.3668 | 1.320e4(NA) | 0.1044 |
|  | ASIAN | 1.416e-14(NA) | >0.9999 | 1.842(0.5251-5.054) | 0.2771 | 7.829e3(NA) | >0.9999 |
|  | AMERICAN INDIAN OR ALASKA NATIVE | 1.02e-18(NA) | >0.9999 | 96.5(5.048-571.5) | **<0.0001** | 9.664e9(NA) | >0.9999 |
| AJCC Pathologic Tumor Stage | Stage I |  |  |  |  |  |  |
|  | Stage II | 0.4094(0.01469-7.123) | 0.5356 | 1.275(0.3306-4.050) | 0.6973 | 1.014e11(NA) | >0.9999 |
|  | Stage III | 0.04801(0.0008193-NA) | 0.0837 | 1.443(0.4949-4.159) | 0.4978 | 3.330e4(NA) | >0.9999 |
|  | Stage IV | 0.1471(0.003572-3.054) | 0.2528 | 2.305(0.6875-7.760) | 0.1755 | 2.359e6(NA) | >0.9999 |
| Histological Type | TPC- Classical/usual |  |  |  |  |  |  |
|  | TPC-Follicular | 0.5974(0.02996-3.894) | 0.6455 | 0.88(0.2563-2.310) | 0.815 | 3.299e-15(NA) | >0.9999 |
|  | TPC-Tall Cell | 1.687e-11(NA) | >0.9999 | 2.756(1.019-6.740) | **0.0331** | 4.393e-10(NA) | >0.9999 |
|  | Other, specify | 6.935e-14(NA) | >0.9999 | 1.873e-11(NA) | 0.9998 | 1.12e-17(NA) | >0.9999 |
| **Tumor Status** | Tumor Free |  |  |  |  |  |  |
|  | With Tumor | 36.09(2.965-1594) | **0.0192** | 16.63(8.283-34.20) | **<0.0001** | 7.893e19(NA) | >0.9999 |
| **PSMA7 CNA** | Diploid |  |  |  |  |  |  |
|  | Amplification | 44.04(1.620-859.0) | **0.0092** | 3.853(0.5991-13.91) | 0.077 | 8.873e4(NA) | 0.0545 |

TCGA, the cancer genome atlas ; THCA, thyroid carcinoma; HR, hazard ratio; OS, overall survival; PFI, progression-free interval;

DSS, disease-specific survival; NA, not available data; AJCC, American Journal of Critical Care; TPC, Thyroid Papillary Carcinoma;

CNA, copy number alteration; *P* value less than 0.05 is shown in bold.

**Table S8. Multivariate Cox regression analysis on the prognosis of TCGA-based KIRC cases.**

| **Variables** | **Group** | **OS** | | **PFI** | | **DSS** | | **DFI** | |
| --- | --- | --- | --- | --- | --- | --- | --- | --- | --- |
|  |  | **HR(95%CI)** | **P value** | **HR(95%CI)** | **P value** | **HR(95%CI)** | **P value** | **HR(95%CI)** | **P value** |
| **Age** | <60years |  |  |  |  |  |  |  |  |
|  | ≥60years | 1.534(1.099-2.163) | **0.013**1 | 1.329(0.9518-1.867) | 0.0969 | 1.197(0.8081-1.788) | 0.3727 | 1.535(0.3380-6.880) | 0.5702 |
| **Gender** | Female |  |  |  |  |  |  |  |  |
|  | Male | 0.8724(0.6202-1.237) | 0.4373 | 1.155(0.7946-1.706) | 0.4578 | 0.9206(0.5962-1.447) | 0.7139 | 1.54(0.2364-9.750) | 0.6401 |
| **Race** | White |  |  |  |  |  |  |  |  |
|  | BLACK OR AFRICAN AMERICAN | 0.883(0.4295-1.618) | 0.71 | 0.7623(0.3534-1.331) | 0.3394 | 0.7174(0.2743-1.548) | 0.444 | 0.2827(0.007652-3.396) | 0.4082 |
|  | ASIAN | 0.7108(0.03984-3.328) | 0.7375 | 0.6975(0.03848-3.445) | 0.7278 | 1.441(0.07781-7.653) | 0.7298 | NA | NA |
| **AJCC Pathologic Tumor Stage** | Stage I |  |  |  |  |  |  |  |  |
|  | Stage II | 1.121(0.5368-2.167) | 0.745 | 2.28(1.036-4.759) | **0.0323** | 3.185(1.110-9.352) | **0.0299** | 1.415(0.1483-9.677) | 0.732 |
|  | Stage III | 1.552(0.9574-2.527) | 0.0749 | 2.307(1.345-4.060) | **0.0029** | 2.907(1.293-7.447) | **0.0154** | 0.4614(0.04324-4.094) | 0.5037 |
|  | Stage IV | 2.389(NA-4.018) | **0.0009** | 3.645(2.154-6.366) | **<0.0001** | 4.712(2.184-11.75) | **0.0002** | NA | NA |
| **Histological Grade** | Grade 1 |  |  |  |  |  |  |  |  |
|  | Grade 2 | 1.204e10(NA) | 0.9997 | 1.765(0.3508-32.15) | 0.5848 | 3.916e11(NA) | >0.9999 | 3.044e8(NA) | >0.9999 |
|  | Grade 3 | 1.582e10(NA) | 0.9997 | 2.632(0.5245-47.90) | 0.3518 | 5.415e11(NA) | >0.9999 | 3.023e8(NA) | >0.9999 |
|  | Grade 4 | 2.212e10(NA) | 0.9997 | 3.213(0.6227-59.02) | 0.2658 | 6.141e11(NA) | >0.9999 | 7.771e7(NA) | >0.9999 |
| **Tumor Status** | Tumor Free |  |  |  |  |  |  |  |  |
|  | With Tumor | 3.31(2.211-4.991) | **<0.0001** | 30.23(16.44-61.12) | **<0.0001** | 2.267e12(NA) | 0.9998 | 51.61(9.960-387.0) | **<0.0001** |
| **PSMA7 CNA** | Diploid |  |  |  |  |  |  |  |  |
|  | Amplification | 0.7983(0.5435-1.154) | 0.2399 | 0.6746(0.4653-0.9663) | **0.0343** | 0.7265(0.4666-1.113) | 0.1486 | 1.783(0.3146-10.23) | 0.5089 |

TCGA, the cancer genome atlas ; KIRC, kidney renal clear cell carcinoma; HR, hazard ratio; OS, overall survival; PFI, progression-free interval;

DSS, disease-specific survival; DFI, disease-free interval; NA, not available data; AJCC, American Journal of Critical Care; CNA, copy number alteration; *P* value less than 0.05 is shown in bold.

**Table S9. Subgroup analysis on the correlation of *PSMA7* expression and prognosis of breast cancer cases.**

| **Factor** | **Subgroup** | **Sample size** | **OS** | | **RFS** | | **DMFS** | |
| --- | --- | --- | --- | --- | --- | --- | --- | --- |
|  |  |  | **HR** | ***P*** | **HR** | ***P*** | **HR** | ***P*** |
| ***ER* status** | *ER* positive | 2565 | 1.64 | **0.0086** | 1.22 | **0.029** | 1.34 | 0.17 |
|  | *ER* negative | 1214 | 0.75 | 0.22 | 0.71 | **0.0029** | 0.76 | 0.28 |
| ***TP53* status** | mutated | 232 | 0.6 | 0.22 | 0.7 | 0.16 | 0.51 | 0.25 |
|  | Wild type | 363 | 3.3 | **0.00017** | 2.28 | **0.017** | 2.9 | **0.0048** |
| ***PR* status** | *PR* positive | 954 | 0.14 | **0.0053** | 1.65 | **0.0044** | 0.25 | **0.042** |
|  | *PR* negative | 1028 | 0.6 | 0.36 | 1.35 | 0.077 | 0.63 | 0.19 |
| ***HER2* status** | *HER2* positive | 416 | 0.8 | 0.22 | 0.66 | 0.13 | 0.48 | 0.092 |
|  | *HER2* negative | 1456 | 0.29 | **0.034** | 1.39 | **0.014** | 0.42 | 0.063 |
| **Grade** | Grade 1 | 378 | 0.45 | 0.086 | 1.96 | **0.011** | 1.48 | 0.48 |
|  | Grade 2 | 1077 | 1.59 | **0.038** | 1.23 | 0.11 | 1.24 | 0.24 |
|  | Grade 3 | 1090 | 0.8 | 0.22 | 0.86 | 0.17 | 0.79 | 0.18 |
| **Intrinsic subtype** | Basal | 879 | 1.23 | 0.4 | 0.71 | **0.024** | 0.68 | 0.13 |
|  | Luminal A | 2504 | 0.56 | **0.0012** | 0.85 | 0.066 | 0.7 | **0.014** |
|  | Luminal B | 1425 | 1.22 | 0.35 | 0.7 | **0.00065** | 0.84 | 0.39 |
|  | *HER2*+ | 335 | 0.52 | 0.052 | 0.59 | **0.0098** | 0.47 | **0.017** |
| **Lymph node status** | Lymph node positive | 1459 | 1.38 | 0.11 | 1.36 | **0.0022** | 0.8 | 0.27 |
|  | Lymph node negative | 2259 | 1.56 | **0.024** | 1.23 | **0.019** | 0.78 | 0.089 |
| **Pietenpol subtype** | Basal-like 1 | 239 | 1.78 | 0.31 | 0.56 | **0.046** | 2.72 | **0.035** |
|  | Basal-like 2 | 97 | 3.85 | **0.024** | 1.53 | 0.32 | 2.65 | **0.044** |
|  | immunomodulatory | 290 | 0.4 | 0.13 | 0.74 | 0.32 | 0.5 | 0.14 |
|  | Mesenchymal | 229 | 0.33 | **0.0039** | 0.48 | **0.00055** | 0.13 | **0.0016** |
|  | Mesenchymal stem-like | 115 | NA | NA | 1.88 | 0.12 | NA | NA |
|  | Luminal androgen receptor | 276 | 0.59 | 0.12 | 0.62 | **0.033** | 0.36 | **0.0099** |

HR, hazard ratio; OS, overall survival; RFS, relapse free survival; DMFS, distant metastasis free survival;

*ER*, Estrogen receptor; *PR*, Progesterone receptor; *HER2*, human epidermal growth factor receptor-2;

*TP53*, Tumor Protein *P53*; NA, not available data; *P* value less than 0.05 is shown in bold.

**Table S10. Subgroup analysis on the correlation of *PSMA7* expression and prognosis of lung cancer cases.**

| **Factor** | **Subgroup** | **Sample size** | **OS** | | **FP** | | **PPS** | |
| --- | --- | --- | --- | --- | --- | --- | --- | --- |
|  |  |  | **HR** | ***P*** | **HR** | **HR** | ***P*** | **HR** |
| **Histology** | adenocarcinoma | 866 | 1.88 | **4.5e-07** | 1.42 | **0.026** | 2.15 | **0.0016** |
|  | squamous cell carcinoma | 675 | 0.87 | 0.27 | 0.38 | **0.00027** | 0.62 | 0.37 |
| **gender** | female | 818 | 1.33 | **0.018** | 1.31 | 0.079 | 1.65 | **0.016** |
|  | male | 1387 | 1.15 | 0.084 | 1.49 | **0.0028** | 1.63 | **0.009** |
| **smoking history** | exclude those never smoked | 970 | 1.38 | **0.0049** | 1.68 | **0.00015** | 1.77 | **0.00022** |
|  | only those never smoked | 247 | 1.92 | **0.021** | 1.28 | 0.31 | 2.09 | **0.026** |
| **stage** | stage I | 652 | 1.67 | **0.00032** | 1.71 | 0.21 | 2.13 | **0.016** |
|  | stage II | 320 | 1.88 | **0.00071** | 1.65 | 0.059 | 2.53 | **0.008** |
|  | stage III | 70 | 1.68 | 0.063 | NA | NA | NA | NA |
| **grade** | grade I | 202 | 1.17 | 0.42 | 1.44 | 0.17 | 2.03 | **0.019** |
|  | grade II | 310 | 1.23 | 0.26 | 0.68 | 0.071 | 1.34 | 0.23 |
|  | grade III | 77 | 2.27 | **0.015** | 2.57 | **0.039** | 6.04 | **0.0024** |
| **AJCC stage t** | t1 | 475 | 1.26 | 0.17 | 0.71 | 0.25 | 0.58 | **0.097** |
|  | t2 | 686 | 0.8 | 0.56 | 1.26 | 0.2 | 1.69 | **0.0032** |
|  | t3 | 99 | 2.14 | **0.0029** | 8.25 | **0.00056** | NA | NA |
|  | t4 | 48 | 0.77 | 0.45 | NA | NA | NA | NA |
| **AJCC stage n** | n0 | 863 | 0.83 | 0.08 | 0.85 | 0.33 | 2.25 | **9.2e-05** |
|  | n1 | 296 | 1.38 | **0.043** | 1.75 | **0.045** | 1.82 | 0.051 |
|  | n2 | 113 | 0.8 | 0.34 | 0.59 | 0.13 | 1.41 | 0.42 |
| **AJCC stage m** | m0 | 818 | 0.92 | 0.4 | 1.5 | 0.13 | 1.66 | 0.11 |
| **surgery** | only surgical margins negative | 730 | 1.54 | **0.00029** | 1.89 | **9.4e-07** | 1.59 | **0.0027** |
| **radiotherapy** | no | 276 | 1.49 | **0.027** | 0.83 | 0.35 | 1.87 | **0.0087** |
|  | yes | 73 | 1.63 | 0.03 | 1.62 | 0.079 | 1.53 | 0.18 |
| **chemotherapy** | no | 317 | 1.28 | 0.21 | 1.25 | 0.27 | 1.87 | **0.015** |
|  | yes | 178 | 1.37 | 0.31 | 1.86 | **0.032** | 1.32 | 0.27 |

HR, hazard ratio; AJCC，American Joint Committee on Cancer; OS, overall survival; FP, first progression; PPS, post progression survival; NA, not available data; *P* value less than 0.05 is shown in bold.

**Table S11. Subgroup analysis on the correlation of *PSMA7* expression and prognosis of ovarian cancer cases.**

| **Factor** | **Subgroup** | **Sample size** | **OS** | | **PFS** | | **PPS** | |
| --- | --- | --- | --- | --- | --- | --- | --- | --- |
|  |  |  | **HR** | ***p*** | **HR** | ***p*** | **HR** | ***p*** |
| **Histology** | Endometrioid | 62 | 3.4e+08 | 0.12 | 0.56 | 0.36 | NA | NA |
|  | Serous | 1232 | 0.82 | **0.017** | 0.83 | **0.017** | 0.75 | **0.003** |
| **Stage** | Stage 1 | 107 | 0.4 | 0.1 | 0.28 | **0.015** | NA | NA |
|  | Stage 2 | 72 | 0.25 | **0.025** | 0.66 | 0.26 | 0.24 | 0.05 |
|  | Stage 3 | 1079 | 0.79 | **0.0094** | 0.86 | 0.067 | 0.72 | **0.0028** |
|  | Stage 4 | 189 | 1.47 | 0.058 | 0.78 | 0.25 | 0.78 | 0.26 |
| **Grade** | Grade 1 | 56 | 0.36 | **0.035** | 0.27 | **0.012** | NA | NA |
|  | Grade 2 | 325 | 0.73 | **0.046** | 0.72 | **0.046** | 0.59 | **0.0093** |
|  | Grade 3 | 1024 | 0.86 | 0.085 | 0.82 | **0.028** | 0.77 | **0.026** |
|  | Grade 4 | 21 | 0.4 | 0.086 | NA | NA | NA | NA |
| ***TP53* mutation** | Mutated | 516 | 0.78 | **0.041** | 0.82 | 0.077 | 0.68 | **0.0048** |
|  | Wild type | 102 | 0.61 | 0.098 | 0.73 | 0.25 | 0.61 | 0.11 |
| **Debulk** | optimal | 802 | 0.69 | **0.0017** | 0.67 | **0.00024** | 0.68 | **0.0062** |
|  | suboptimal | 536 | 0.81 | 0.064 | 0.7 | **0.001** | 0.86 | 0.26 |
| **Chemotherapy** | Contains platin | 1438 | 0.85 | **0.04** | 0.8 | **0.00081** | 0.79 | **0.018** |
|  | Contains Taxol | 821 | 0.82 | **0.044** | 0.79 | **0.0077** | 0.83 | 0.084 |
|  | Contains Taxol+platin | 804 | 0.82 | **0.038** | 0.77 | **0.0051** | 0.83 | 0.095 |
|  | Contains Avastin | 50 | 0.3 | **0.012** | 0.65 | 0.23 | 0.3 | **0.011** |
|  | Contains Docetaxel | 108 | 0.64 | 0.11 | 1.35 | 0.24 | 0.65 | 0.15 |
|  | Contains Gemcitabine | 135 | 0.68 | 0.056 | 0.56 | **0.0065** | 0.63 | **0.043** |
|  | Contains Paclitaxel | 248 | 1.45 | 0.11 | 1.62 | **0.023** | 0.69 | 0.2 |
|  | Contains Topotecan | 119 | 0.71 | 0.12 | 0.59 | **0.017** | 0.64 | 0.064 |

HR, hazard ratio; OS, overall survival; PFS, progress free survival; PPS, post progression survival;

*TP53*, Tumor Protein *P53*; NA, not available data; *P* value less than 0.05 is shown in bold.

**Table S12. Subgroup analysis on the correlation of *PSMA7* expression and prognosis of gastric cancer cases.**

| **Factor** | **Subgroup** | **Sample size** | **OS** | | **FP** | | **PPS** | |
| --- | --- | --- | --- | --- | --- | --- | --- | --- |
|  |  |  | **HR** | ***P*** | **HR** | ***P*** | **HR** | ***P*** |
| **Gender** | Female | 244 | 1.72 | **0.012** | 2.27 | **0.0017** | 1.93 | **0.042** |
|  | Male | 567 | 1.17 | 0.21 | 1.44 | **0.03** | 1.18 | 0.36 |
| **Stage** | Stage 1 | 69 | 4.65 | **0.029** | 8.46 | **0.016** | NA | NA |
|  | Stage 2 | 145 | 0.36 | **0.015** | 0.26 | **0.0058** | NA | NA |
|  | Stage 3 | 319 | 1.35 | 0.081 | 1.72 | **0.033** | 1.75 | 0.086 |
|  | Stage 4 | 152 | 0.6 | 0.11 | 1.56 | 0.11 | 0.5 | 0.12 |
| **Stage t** | t2 | 253 | 0.51 | 0.061 | 0.51 | 0.058 | 0.51 | 0.12 |
|  | t3 | 208 | 1.75 | **0.025** | 1.66 | **0.035** | 1.78 | 0.05 |
| **Stage n** | n0 | 76 | 0.6 | 0.34 | 0.58 | 0.28 | NA | NA |
|  | n1 | 232 | 0.67 | 0.13 | 0.59 | 0.092 | 0.52 | **0.048** |
|  | n2 | 129 | 1.98 | 0.072 | 1.78 | 0.12 | 1.58 | 0.21 |
|  | n3 | 76 | 0.31 | **0.03** | 0.18 | **0.005** | NA | NA |
| **Stage m** | m0 | 459 | 0.76 | 0.16 | 1.34 | 0.18 | 0.78 | 0.28 |
|  | m1 | 58 | 0.8 | 0.61 | 1.33 | 0.54 | NA | NA |
| ***HER2*** | negative | 641 | 1.51 | **0.0029** | 1.84 | **0.00051** | 0.81 | 0.37 |
|  | positive | 425 | 0.79 | 0.14 | 1.45 | **0.036** | 1.28 | 0.22 |
| **Lauren classification** | Instestinal | 336 | 1.27 | 0.24 | 1.43 | 0.16 | 0.8 | 0.46 |
|  | Diffuse | 248 | 0.75 | 0.26 | 0.73 | 0.22 | 1.49 | 0.27 |
|  | Mixed | 33 | 0.46 | 0.31 | 6.49 | **0.049** | NA | NA |
| **Differentiation** | Poorly | 166 | 1.32 | 0.17 | 1.48 | 0.091 | 1.73 | 0.11 |
|  | Moderately | 67 | 1.49 | 0.25 | 1.65 | 0.14 | 0.69 | 0.45 |
|  | Well | 32 | 3.99 | **0.045** | NA | NA | NA | NA |
| **Treatment** | Surgery alone | 393 | 0.68 | 0.064 | 0.76 | 0.16 | 0.63 | 0.072 |
|  | 5-Fu based adjuvant | 158 | 2.62 | **9.0e-07** | 2.92 | **4.9e-08** | 1.4 | 0.1 |
| **Perforation** | No | 169 | 1.29 | 0.28 | 1.32 | 0.21 | 0.75 | 0.36 |

HR, hazard ratio; OS, overall survival; FP, first progression; PPS, post progression survival;

*HER2*, human epidermal growth factor receptor-2; NA, not available data; *P* value less than 0.05 is shown in bold.

**Table S13. Subgroup analysis on the correlation of *PSMA7* expression and prognosis of liver cancer cases.**

| **Factor** | **Subgroup** | **Sample size** | **OS** | | **PFS** | | **RFS** | | **DSS** | |
| --- | --- | --- | --- | --- | --- | --- | --- | --- | --- | --- |
|  |  |  | **HR** | ***P*** | **HR** | ***P*** | **HR** | ***P*** | **HR** | ***P*** |
| **Stage** | Stage 1 | 171 | 1.55 | 0.16 | 0.66 | 0.12 | 1.63 | 0.12 | 0.48 | 0.18 |
|  | Stage 2 | 86 | 0.68 | 0.33 | 0.6 | 0.19 | 0.7 | 0.39 | 1.68 | 0.36 |
|  | Stage 3 | 85 | 0.64 | 0.14 | 1.66 | 0.071 | NA | NA | 0.54 | 0.13 |
| **Grade** | Grade 1 | 55 | 0.45 | 0.1 | 1.67 | 0.19 | NA | NA | 0.36 | 0.083 |
|  | Grade 2 | 177 | 0.71 | 0.33 | 1.34 | 0.25 | 1.63 | 0.088 | 0.59 | 0.27 |
|  | Grade 3 | 122 | 1.78 | 0.065 | 0.63 | 0.091 | 1.56 | 0.11 | 1.95 | 0.079 |
| **AJCC_T** | T1 | 181 | 1.45 | 0.21 | 0.7 | 0.17 | 1.62 | 0.11 | 0.55 | 0.14 |
|  | T2 | 94 | 1.42 | 0.36 | 0.68 | 0.27 | 1.43 | 0.26 | 2.08 | 0.16 |
|  | T3 | 80 | 0.62 | 0.13 | 1.68 | 0.067 | 1.59 | 0.14 | 1.58 | 0.22 |
| **Gender** | Female | 121 | 2.35 | **0.023** | 1.26 | 0.4 | 1.88 | 0.058 | 1.98 | 0.08 |
|  | Male | 250 | 1.37 | 0.17 | 0.82 | 0.27 | 1.18 | 0.42 | 0.7 | 0.24 |
| **Vascular invasion** | None | 205 | 1.39 | 0.23 | 0.74 | 0.19 | 1.44 | **0.2** | 0.72 | 0.36 |
|  | micro | 93 | 2.8 | 0.081 | 1.76 | **0.049** | 2.07 | **0.024** | 3.1e+08 | **0.021** |
| **Race** | White | 184 | 1.67 | **0.03** | 1.36 | 0.13 | 1.69 | **0.021** | 1.95 | **0.021** |
|  | Asian | 158 | 0.6 | 0.092 | 0.6 | **0.042** | 0.63 | 0.081 | 0.43 | **0.033** |
| **Sorafenib treatment** | treated | 30 | 3.7 | 0.073 | 1.63 | 0.26 | 2.44 | 0.064 | 3.7 | 0.073 |
| **Alcohol consumption** | Yes | 117 | 0.66 | 0.23 | 0.64 | 0.11 | 0.71 | 0.29 | 0.5 | 0.06 |
|  | none | 205 | 1.53 | 0.14 | 0.82 | 0.35 | 1.56 | 0.12 | 1.9 | 0.097 |
| **Hepatitis virus** | Yes | 153 | 1.78 | 0.081 | 0.71 | 0.16 | 0.72 | 0.29 | 1.63 | 0.3 |
|  | none | 169 | 1.36 | 0.18 | 1.39 | 0.15 | 1.69 | **0.039** | 1.29 | 0.41 |

HR, hazard ratio; AJCC，American Joint Committee on Cancer; OS, overall survival; PFS, progress free survival;

RFS, relapse free survival; DSS, disease specific surviva; NA, not available data; *P* value less than 0.05 is shown in bold.

**Table S14. COSMIC-annotated CNA type of *PSMA7* in cell lines tested with compounds in GDSC/CTRP databases.**

| **COSMIC** | | | | | | | | **GDSC** | | | | | **CTRP** | |  |
| --- | --- | --- | --- | --- | --- | --- | --- | --- | --- | --- | --- | --- | --- | --- | --- |
| **Cell Line** | **Cell Line ID** | **Expression** | **CNA Type** | **CN** | **CNA Segment Posn.** | **Average Ploidy** | **CNA Id** | **LN IC50** | **IC50(uM)** | | | **AUC** | **AUC sensitivity score** | |  |
| **Candidate resistant compound: TPCA-1** | | | | | | | | | | | | | | |  |
| Caov-4 | 949090 | over | gain | 9 | 20:61037483..64324801 | 4.639966845 | 232713 | 5.826597 | 339.202407 | | | 0.968947 | / | |  |
| ChaGo-K-1 | 687596 | over | gain | 5 | 20:58858046..62479806 | 2.13539469 | 151310 | 4.52658 | 92.44186866 | | | 0.950963 | 13.953 | |  |
| HT-144 | 907067 | over | gain | 5 | 20:56127356..64324801 | 2.661321364 | 261529 | 4.801902 | 121.7417503 | | | 0.95678 | 14.426 | |  |
| JIMT-1 | 1298157 | over | gain | 5 | 20:61688729..64324801 | 2.510993088 | 271615 | 3.943614 | 51.60476432 | | | 0.933545 | 13.651 | |  |
| KYSE-520 | 753575 | over | gain | 5 | 20:38934669..64324801 | 2.692340388 | 15319 | 4.919434 | 136.9250916 | | | 0.95293 | 16.976 | |  |
| MDA-MB-361 | 908121 | over | gain | 5 | 20:60604213..63004093 | 2.654787781 | 109582 | 4.309878 | 74.43140775 | | | 0.955889 | 18.743 | |  |
| NCI-H2085 | 687812 | normal | gain | 5 | 20:58380008..62577441 | 2.672741274 | 293419 | 2.452956 | 11.62265255 | | | 0.81047 | 11.637 | |  |
| NCI-H2444 | 1298356 | over | gain | 5 | 20:49477821..64324801 | 2.361224437 | 206579 | 4.889023 | 132.8237418 | | | 0.966881 | 14.313 | |  |
| SJRH30 | 909716 | over | gain | 9 | 20:61719595..63696229 | 4.66488877 | 180386 | 4.206984 | 67.15369852 | | | 0.960052 | 12.52 | |  |
| **Candidate sensitive compound: Docetaxel** | | | | | | | | | | | | | | |  |
| Caov-4 | 949090 | over | gain | 9 | 20:61037483..64324801 | 4.639966845 | 232713 | -6.849656 | | 0.00105982 | | 0.5472 | | / | |
| ChaGo-K-1 | 687596 | over | gain | 5 | 20:58858046..62479806 | 2.13539469 | 151310 | -6.084023 | | 0.00227899 | | 0.670955 | | 7.4812 | |
| HT-144 | 907067 | over | gain | 5 | 20:56127356..64324801 | 2.661321364 | 261529 | -5.487183 | 0.004139489 | | | 0.749881 | 10.418 | | |
| JIMT-1 | 1298157 | over | gain | 5 | 20:61688729..64324801 | 2.510993088 | 271615 | -4.947352 | 0.007102191 | | | 0.807974 | / | | |
| KYSE-520 | 753575 | over | gain | 5 | 20:38934669..64324801 | 2.692340388 | 15319 | -4.902798 | 0.007425777 | | | 0.800852 | 8.0548 | | |
| MDA-MB-361 | 908121 | over | gain | 5 | 20:60604213..63004093 | 2.654787781 | 109582 | -4.708826 | 0.009015355 | | | 0.810737 | 11.402 | | |
| NCI-H2085 | 687812 | over | gain | 5 | 20:58380008..62577441 | 2.672741274 | 293419 | -4.28957 | | | 0.01371082 | 0.864832 | / | | |
| NCI-H2444 | 1298356 | over | gain | 5 | 20:49477821..64324801 | 2.361224437 | 206579 | -3.730548 | | | 0.023979691 | 0.912316 | **/** | | |
| SJRH30 | 909716 | over | gain | 9 | 20:61719595..63696229 | 4.66488877 | 180386 | -1.612761 | | | 0.199336486 | 0.967644 | **/** | | |

COSMIC, catalogue of somatic mutations in cancer; GDSC, genomics drug sensitivity in cancer; CTRP, cancer therapeutics response portal; CN, copy number; CNA, copy number alteration; IC50, half maximal inhibitory concentration; AUC, area under the curve.

**Supplementary Figures**

**
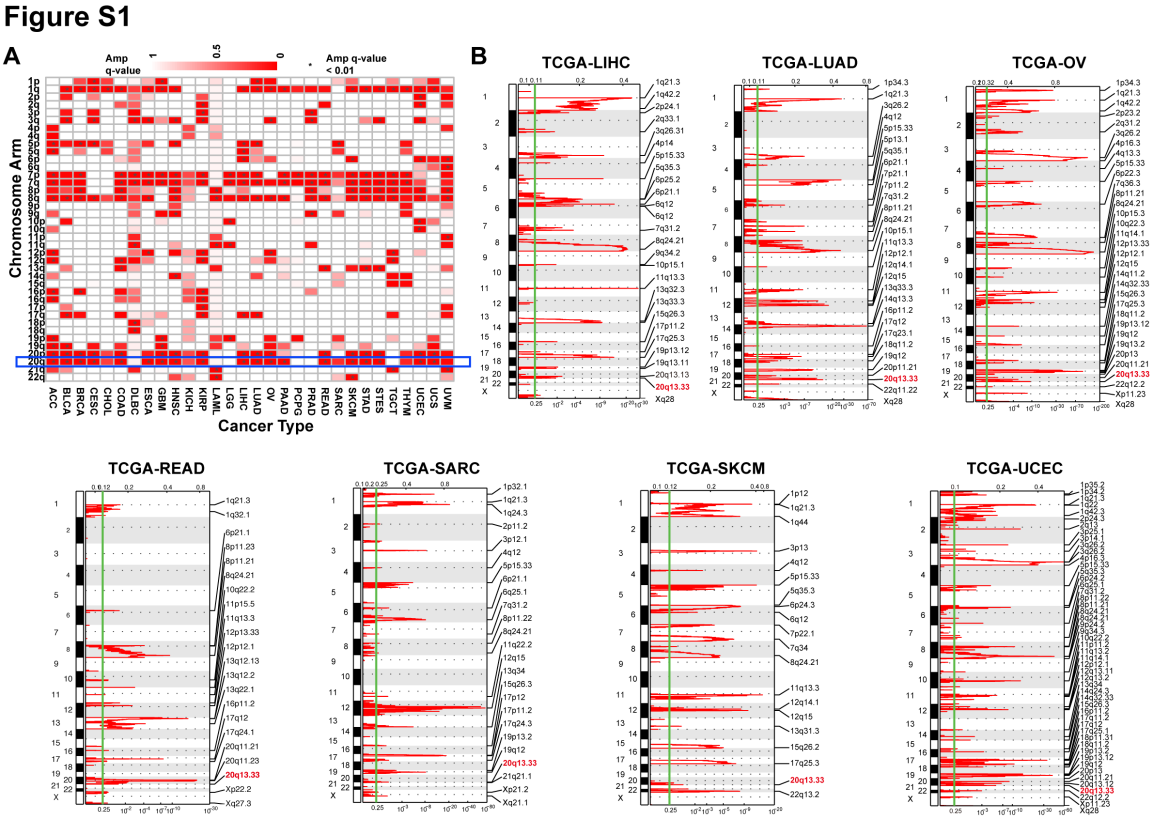
Figure S1**. Genomic amplification of chromosome 20q across TCGA tumors. Using the SNP6 copy number analysis (GISTIC2) from Broad Firehose, (**A**) the statistical data for each chromosome arm across the whole genome in 30 types of TCGA tumors was obtained and shown in a heatmap with a spotlight on the 20q arm. (**B**) Among the 7 types of cancers, the detailed plots for focal amplification of 20q13.33 were displayed.

**
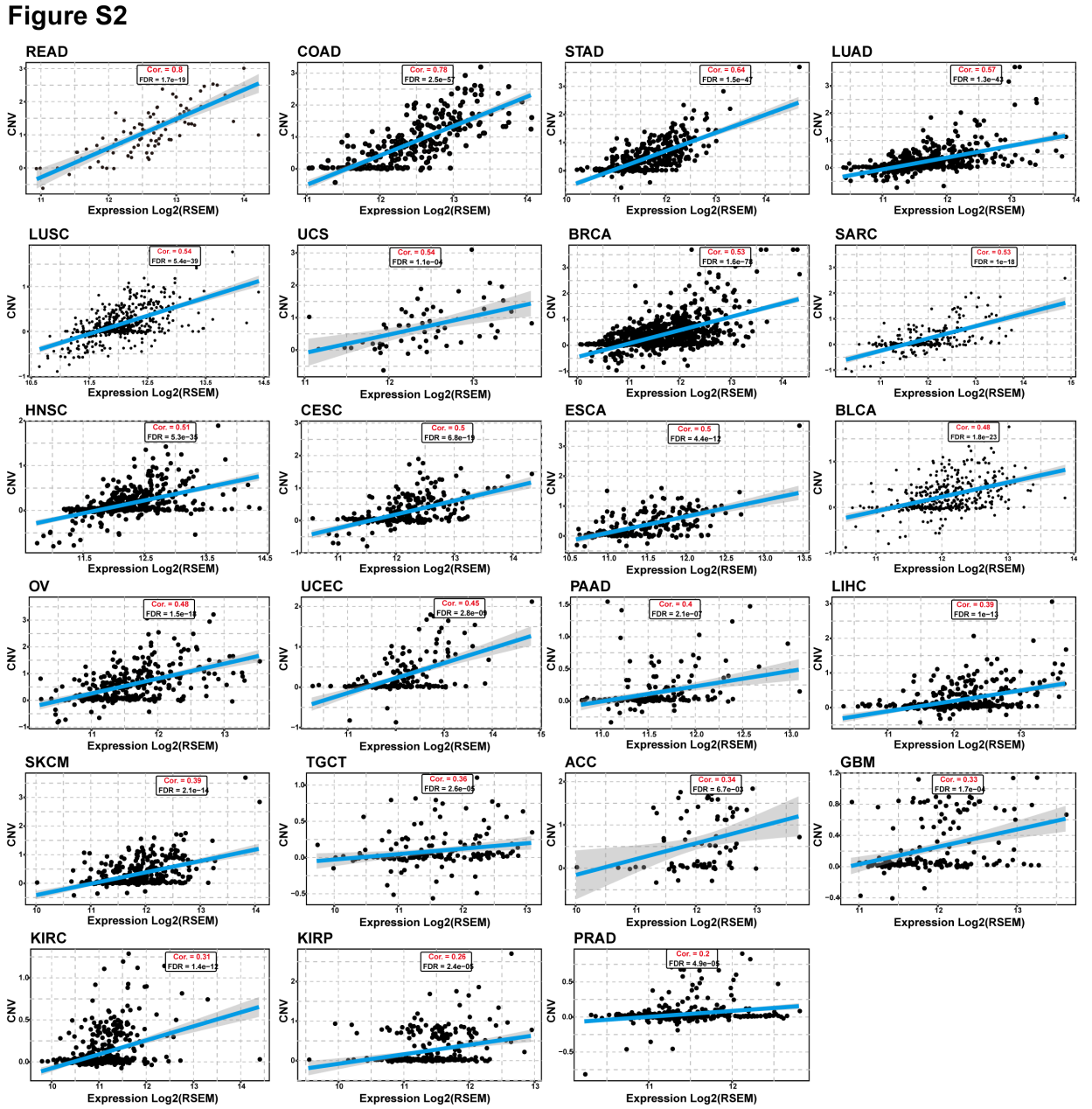
Fig. S2.** The correlations between *PSMA7* copy number alteration (CNA) and expression across TCGA tumors. Based on 33 types of TCGA tumors, the potential association between CNA and *PSMA7* mRNA expression was investigated. The detailed data were shown as scatter plots.

**
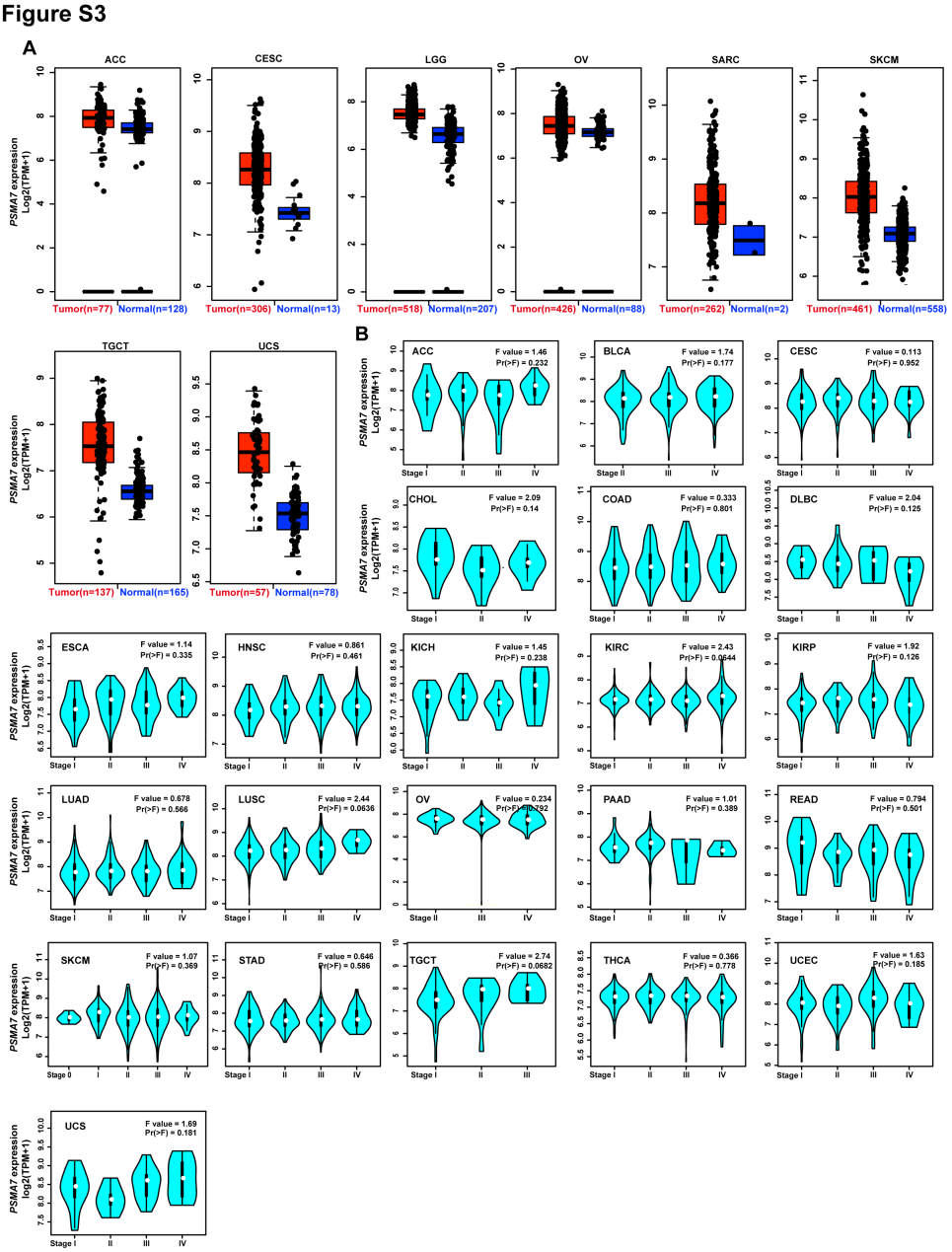
Fig. S3.** Expression level of *PSMA7* in various tumors and pathological stages. (**A**) The expression level of *PSMA7* in ACC, CESC, LGG, OV, SARC, SKCM, TGCT, and UVM of TCGA tumors was analyzed through GEPIA2, with the matched normal tissues of the GTEx dataset added as controls. The results were presented as a box plot. (**B**) The expression of *PSMA7* in different pathological stages of BRCA and LIHC in the TCGA cohorts was evaluated by GEPIA2, with Log2 (TPM + 1) applied for the log-scale.

**
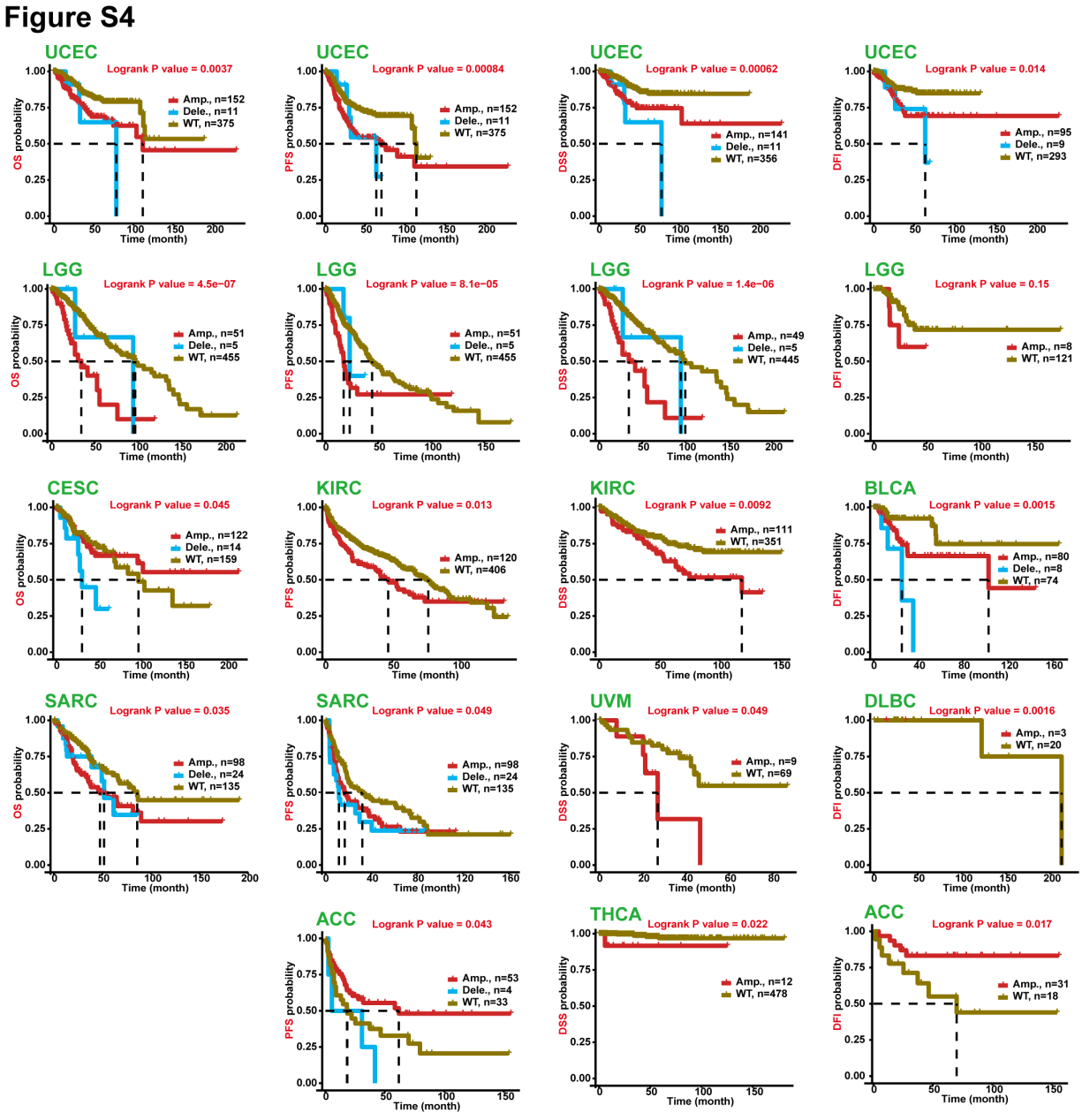
Fig. S4.** The K-M survival curve for *PSMA7* CNA in detailed types of TCGA tumors. The GSCA web was applied to explore the potential association between *PSMA7* CNA and OS, PFS, DSS, and DFI survival prognosis for TCGA tumors. The data with log-rank P values less than 0.05 were displayed.

**
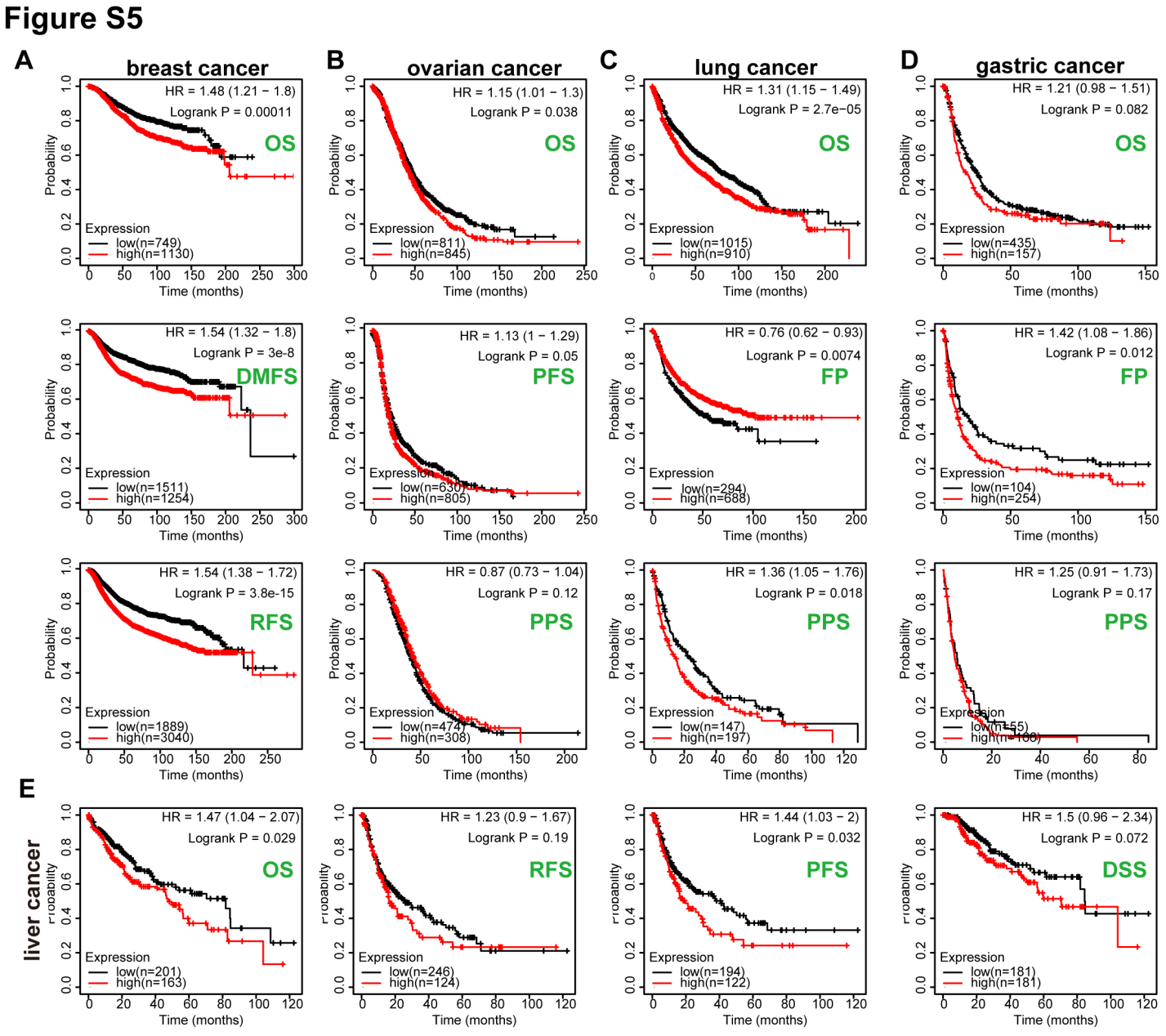
Fig. S5.** Correlation between *PSMA7* gene expression and prognosis of cancers using the Kaplan-Meier plotter. The Kaplan-Meier plotter was used to perform a series of survival analyses, including OS, DMFS, RFS, PFS, PPS, FP, and DSS, by the expression level of *PSMA7* in breast cancer (**A**), ovarian cancer (**B**), lung cancer (**C**), gastric cancer (**D**), and liver cancer (**E**) cohorts.

**
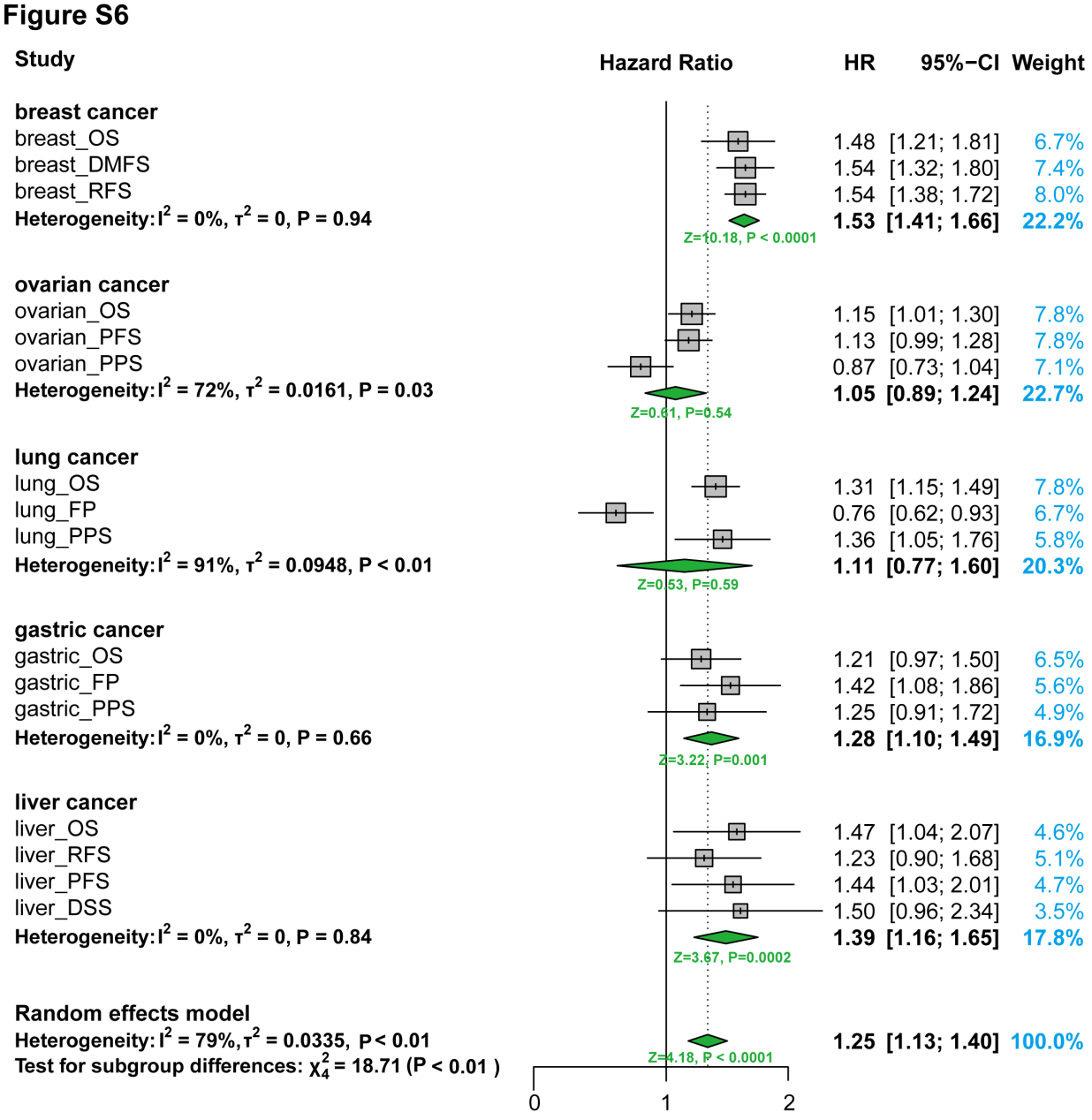
Fig. S6.** Meta-analysis on the correlation between *PSMA7* expression and cancer prognosis. A meta-analysis was performed for the pooling of a series of survival analyses via the expression level of *PSMA7* in breast cancer, ovarian cancer, lung cancer, gastric cancer, and liver cancer patients.

**
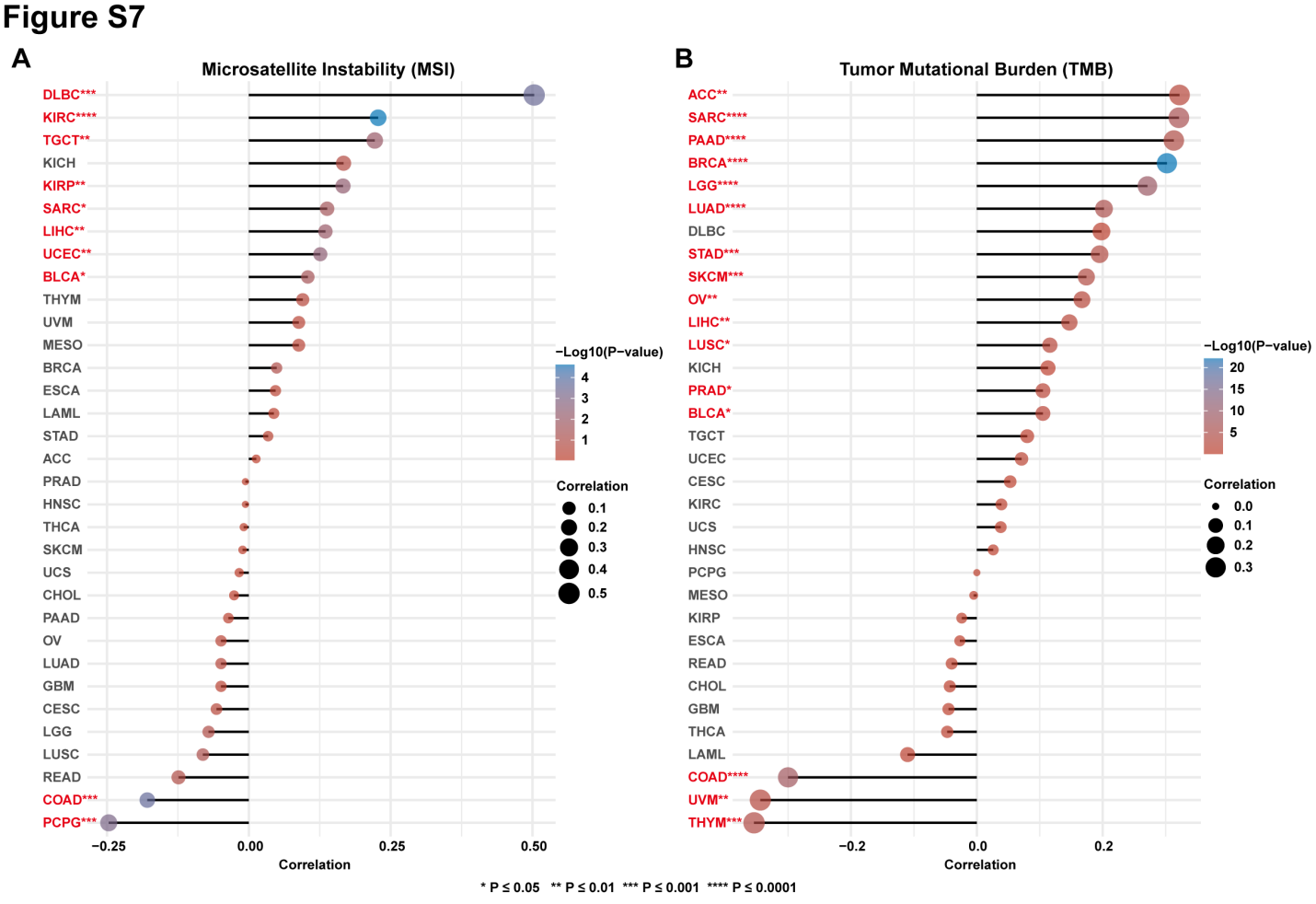
Fig. S7.** Correlation between *PSMA7* expression and tumor mutational burden /microsatellite instability. Based on tumor data from TCGA, the potential association of *PSMA7* expression with (**A**) tumor mutational burden (TMB) and (**B**) microsatellite instability (MSI) was investigated by the Assistant for Clinical bioinformatics tool, respectively. Both the partial correlation (cor) values and P-value are attached. * P ≤ 0.05; ** P ≤ 0.01; *** P ≤ 0.001; **** P ≤ 0.0001.


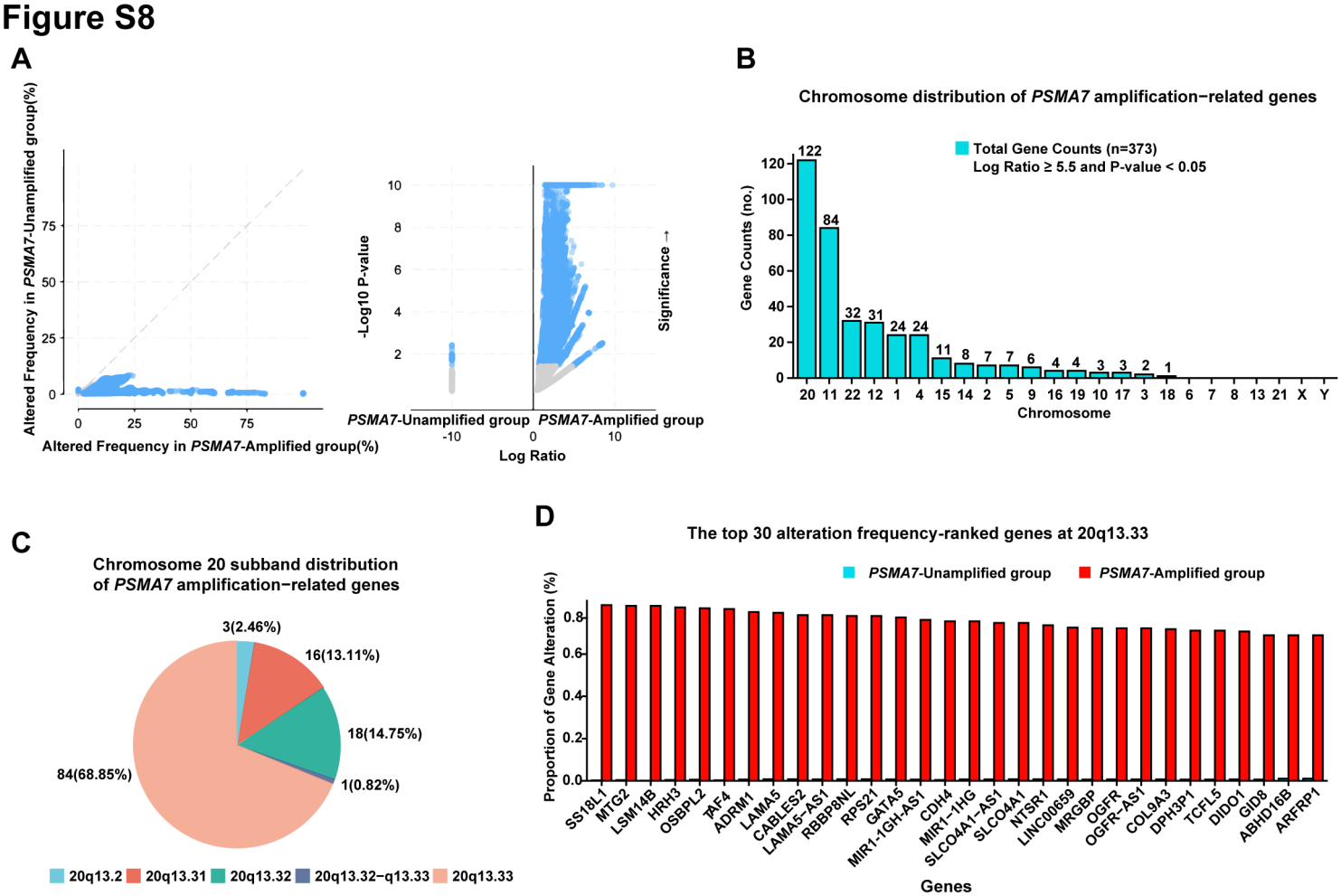


**Fig. S8.** The co-occurrence amplification pattern of *PSMA7* and other genes at 20q in TCGA tumors. (**A**) Based on cases with CNA-amplified *PSMA7* (n=276) and those without the corresponding abnormality (n=10684) in TCGA tumors, the cBioPortal tool was used to explore the differential genes with the statistically significant alteration frequency. The data was visualized as a scatter diagram and volcano plot. The genes with alteration frequency of more than 5.5 log ratio, were focused on and explored their enriched regions at chromosome (**B**) and chromosome subband (**C**). The top 30 frequency-ranked genes located at 20q13.33, were displayed (**D**).

**
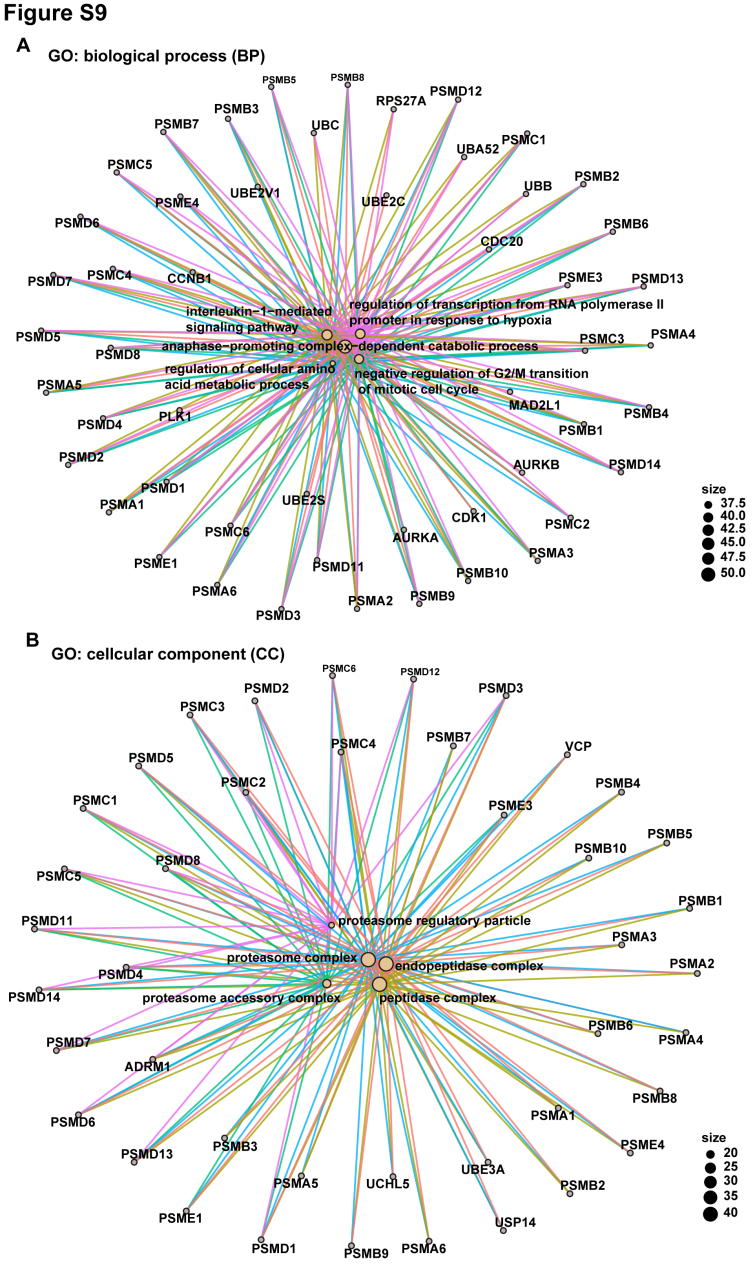
Fig. S9.** GO biological process/cellular component analysis of *PSMA7*-related genes in tumors. Based on the PSMA7-binding and correlated genes, GO enrichment analysis was performed. The cnetplots for the cellular component (**A**) and biological process (**B**) data were displayed.

**
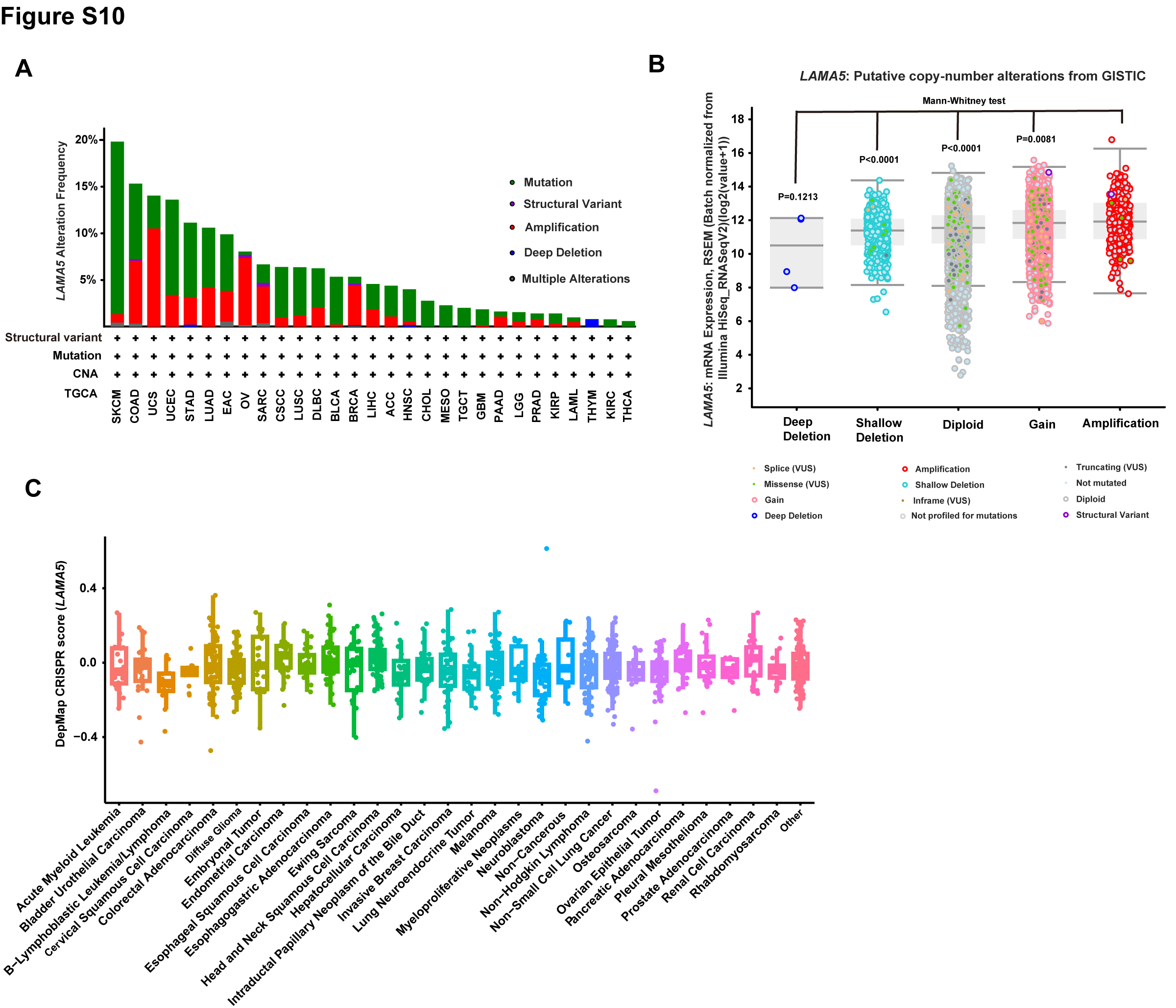
Figure S10.** The genetic alteration, gene expression, and cell dependency of *LAMA5* in tumors. (**A**) The genetic alteration type and frequency of *LAMA5* were analyzed by the cBioPortal across 32 types of TCGA tumors. (**B**) Based on the copy number data of GISTIC databases, the cBioPortal was applied to estimate the expression of *LAMA5* in different types of putative. (**C**) The effect of the *LAMA5* depletion on tumor cell survival was evaluated by the large-scale CRISPR knockout screens in the 1095 cancer cell lines from the Depmap database.
